# Melanocortin 1 receptor mediates melanin production by interacting with the BBSome in primary cilia

**DOI:** 10.1371/journal.pbio.3002940

**Published:** 2024-12-02

**Authors:** Xiaoyu Tian, Hanyu Wang, Song Liu, Wei Liu, Kaiyue Zhang, Xiaohan Gao, Qingchao Li, Huijie Zhao, Liangran Zhang, Peiwei Liu, Min Liu, Youjun Wang, Xueliang Zhu, Rutao Cui, Jun Zhou

**Affiliations:** 1 Center for Cell Structure and Function, Shandong Provincial Key Laboratory of Animal Resistance Biology, College of Life Sciences, Shandong Normal University, Jinan, China; 2 Department of Pediatric Surgery, Shandong Provincial Hospital Affiliated to Shandong First Medical University, Jinan, China; 3 Key Laboratory of Cell Proliferation and Regulation Biology of the Ministry of Education, College of Life Sciences, Beijing Normal University, Beijing, China; 4 State Key Laboratory of Cell Biology, Shanghai Institute of Biochemistry and Cell Biology, Center for Excellence in Molecular Cell Science, Chinese Academy of Sciences, Shanghai, China; 5 Skin Disease Research Institute, The 2nd Hospital, Zhejiang University, Hangzhou, China; 6 Department of Genetics and Cell Biology, State Key Laboratory of Medicinal Chemical Biology, Haihe Laboratory of Cell Ecosystem, College of Life Sciences, Nankai University, Tianjin, China; Rheinische Friedrich-Wilhelms-Universitat Bonn, GERMANY

## Abstract

Production of melanin pigments is a protective mechanism of the skin against ultraviolet (UV)-induced damage and carcinogenesis. However, the molecular basis for melanogenesis is still poorly understood. Herein, we demonstrate a critical interplay between the primary cilium and the melanocortin 1 receptor (MC1R) signaling. Our data show that UV and α-melanocyte-stimulating hormone (α-MSH) trigger cilium formation in human melanocytes and melanoma cells. Deficiency of MC1R or the presence of its red hair color (RHC) variations significantly attenuates the UV/α-MSH-induced ciliogenesis. Further investigation reveals that MC1R enters the cilium upon UV/α-MSH stimulation, which is facilitated by the interaction of MC1R with the BBSome and the palmitoylation of MC1R. MC1R interacts with the BBSome through the second and third intercellular loops, which contain the common RHC variant alleles (R151C and R160W). These RHC variants of MC1R exhibit attenuated ciliary localization, and enforced ciliary localization of these variants elevates melanogenesis. Ciliary MC1R triggers a sustained cAMP signaling and selectively stimulates Sox9, which appears to up-regulate melanogenesis-related genes as the transcriptional cofactor for MITF. These findings reveal a previously unrecognized nexus between MC1R and cilia and suggest an important mechanism for RHC variant-related pigmentary defects.

## Abstract 2

Melanin production in the skin protects us against UV damage, but how? These authors show that upon UV exposure MC1R enters primary cilia and interacts with the BBSome, triggering the downstream signalling that stimulates melanogenesis. Red-haired people have a genetic variation in MC1R that affects this interaction, leading to defective melanin production.

## Introduction

Skin color is determined by the content of melanin in the epidermis, which correlates with ultraviolet (UV) response and pigmentary diseases [1]. Melanin protects the skin from UV radiation, which leads to increased production of free radicals and hence causes skin damage, skin inflammation, premature skin aging, and skin cancer [2]. Thus, fair-skinned people have an increased risk for UV-stimulated skin damage and skin cancer compared to darker-skinned people [1]. Failure in melanocyte activity, melanin synthesis, or melanosome transport leads to pigmentation-related disorders, such as oculocutaneous albinism, vitiligo, Hermansky–Pudlak syndrome, and Griscelli syndrome [1]. These pigmentary diseases not only reduce the quality of life, but also increase the risk of other skin diseases, including skin cancer.

Melanocytes are known to form primary cilia both in vitro and in vivo [3–5]. The primary cilium is an organelle protruding from the surface of most vertebrate cells and plays a critical role in signal sensing and transduction [6,7]. The fundamental structure of the cilium contains a basal body derived from the mother centriole, a microtubule-based core known as the axoneme, and a ciliary membrane covering the axoneme [8]. Recent studies show that physically sequestering signaling pathways to either the cilium or the cell body allows parallel information processing without crosstalk, while more evidence is needed to explain how cells distinguish ciliary from extraciliary cAMP to convey different information [9,10]. A number of G protein-coupled receptors (GPCRs) are known to localize to the ciliary membrane, including melanocortin 4 receptor (MC4R) [11–13]. GPCR proteins are transported across the transition zone barrier into or out of the cilium with the help of intraflagellar transport (IFT) trains and adaptor proteins, such as a complex composed of 8 Bardet–Biedl syndrome (BBS) proteins known as the BBSome [14,15]. A recent study has demonstrated a ciliary regulation of MC4R by its associated protein, melanocortin 2 receptor accessory protein 2 (MRAP2) [16]. The functions and mechanisms of other melanocortin receptors and their ciliary regulation remain to be demonstrated.

Melanocortin 1 receptor (MC1R) is a GPCR that transduces signals during tanning and pigmentation in humans and mice [17]. In melanocytes, MC1R triggers an increase in the cAMP level upon stimulation by its ligand, α-melanocyte-stimulating hormone (α-MSH) [18]. Through the protein kinase A (PKA) signaling and interaction with **cAMP-response element** binding protein (CREB), MC1R regulates the production of microphthalmia-associated transcription factor (MITF), which subsequently triggers the expression of a large portion of melanogenesis-related genes. Red hair color (RHC) variants of MC1R are associated with phenotypes such as red or blonde hair, fair skin, freckling, and skin sensitivity to UV radiation [19]. RHC variants exhibit a reduced ability to trigger melanogenesis and an increased correlation with melanoma, and thus exploring their pathogenetic mechanisms would greatly benefit therapeutic progress [19]. The MC1R signaling is also known to be regulated by posttranslational modifications, such as palmitoylation [20,21]. However, the molecular details and regulatory mechanisms underlying MC1R signaling still need further investigation. In the present study, we demonstrate significant cilium formation during the initiation of melanogenesis. Mechanistic studies reveal that MC1R is located to the cilium with the help of the BBSome, in a palmitoylation-dependent manner. MC1R interacts with the BBSome through BBS2 and the second/third intercellular loops of MC1R, respectively. Our data also show that ciliary MC1R stimulates a robust cAMP signaling in melanogenesis and selectively up-regulates Sox9, which may be responsible for the sustained tanning process in UV-triggered skin pigmentation. The attenuated ciliogenesis, BBSome binding, and ciliary localization ability of MC1R RHC variants may suggest potential mechanisms for RHC-related diseases.

## Results

### UV and α-MSH trigger ciliogenesis in melanocytes and melanoma cells

**Fig 1:**
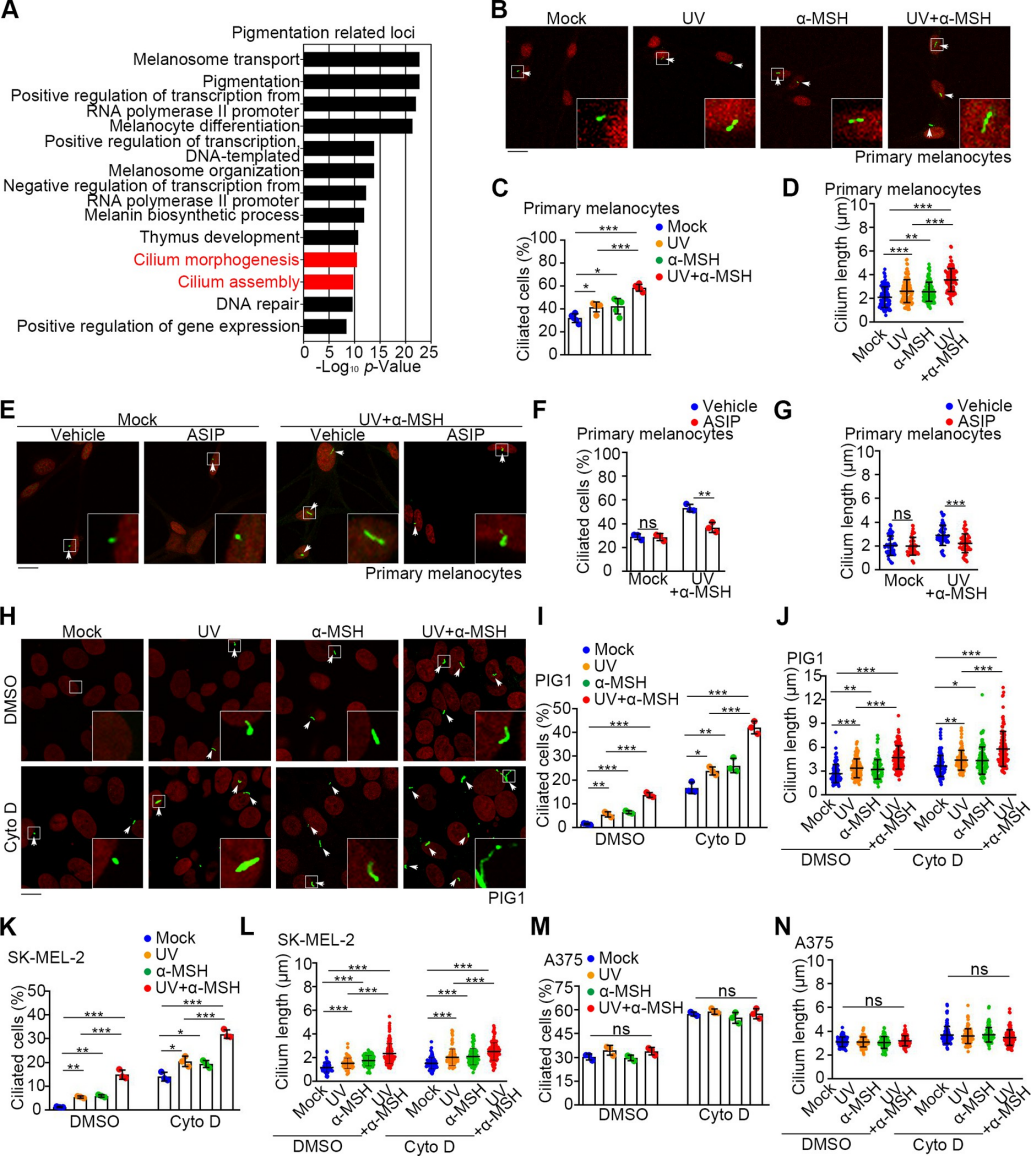
UV and α-MSH induce cilium formation in melanocytes and melanoma cells. (A) Top 13 enriched terms in the GO analysis of pigmentary genes listed in the International Federation of Pigment Cell Societies. (B–D) Immunofluorescence images (B) and quantification of the percentage of ciliated cells (*n* = 5 independent experiments) (C) and cilium length (*n* ≥ 50 cells) (D) of primary human melanocytes that were mock-treated, or treated with UV, 100 nM α-MSH, or both. Cells were treated with α-MSH for 24 h in the presence of serum after UV exposure. Cells were stained with the Arl13b antibody (green) and DAPI (red, pseudocolor). Ciliated cells were marked with white arrows. Scale bar, 10 μm. (E–G) Immunofluorescence images (E) and quantification of the percentage of ciliated cells (*n* = 3 independent experiments) (F) and cilium length (*n* ≥ 50 cells) (G) of primary human melanocytes that were treated with vehicle (PBS) or 10 nM ASIP with or without UV/α-MSH (100 nM). Cells were treated with α-MSH for 24 h in the presence of serum after UV exposure. Cells were stained with the Arl13b antibody (green) and DAPI (red, pseudocolor). Ciliated cells were marked with white arrows. Scale bar, 10 μm. (H–J) Immunofluorescence images (H) and quantification of the percentage of ciliated cells (*n* = 3 independent experiments) (I) and cilium length (*n* ≥ 50 cells) (J) of PIG1 cells that were mock-treated, or treated with UV, 100 nM α-MSH, or both. Cells were treated with α-MSH for 24 h in the absence of serum after UV exposure, under either vehicle (DMSO) or 100 nM cytochalasin D (Cyto D) treatment conditions. Cells were stained with the Arl13b antibody (green) and DAPI (red, pseudocolor). Ciliated cells were marked with white arrows. Scale bar, 10 μm. (K and L) Quantification of the percentage of ciliated cells (*n* = 3 independent experiments) (K) and cilium length (*n* ≥ 50 cells) (L) of SK-MEL-2 cells that were mock-treated, or treated with UV, 100 nM α-MSH, or both. Cells were treated with α-MSH for 24 h in the absence of serum after UV exposure, under either vehicle (DMSO) or Cyto D treatment conditions. Cells were stained with the Arl13b antibody (green) and DAPI (red, pseudocolor). Scale bar, 10 μm. (M and N) Quantification of the percentage of ciliated cells (*n* = 3 independent experiments) (M) and cilium length (*n* ≥ 50 cells) (N) of A375 cells that were mock-treated, or treated with UV, 100 nM α-MSH, or both. Cells were treated with α-MSH for 24 h in the absence of serum after UV exposure, under either vehicle (DMSO) or Cyto D treatment conditions. Cells were stained with the Arl13b antibody (green) and DAPI (red, pseudocolor). Scale bar, 10 μm. Data are presented as mean ± SD. Statistical significance was determined by one-way ANOVA (C, D, and I–N) or unpaired two-tailed Student’s *t* test (F and G); *p < 0.05, **p < 0.01, ***p < 0.001; ns, not significant. See also S1 Fig. The underlying data for this figure can be found in S1 File and S1 Data. α-MSH, α-melanocyte-stimulating hormone; ASIP, agouti-signaling protein; GO, gene ontology; UV, ultraviolet.

To investigate the molecular mechanisms regulating melanogenesis, we performed gene ontology (GO) analysis for all the pigmentary genes listed in the International Federation of Pigment Cell Societies. As expected, signaling pathways related to the melanocyte behavior and transcriptional regulation were enriched among the top classified terms, while there was also enrichment in cilium-related pathways (Fig 1A). We also compared the transcriptome of sun-exposed skins from the Gene Expression Omnibus (GEO GSE56754) and found a widespread increase of cilium-related genes after UVB treatment (S1A Fig). These results suggest a connection between melanogenesis and the primary cilium.

To examine the temporal relationship between ciliogenesis and melanogenesis, primary human melanocytes, which exhibit robust ciliogenesis upon serum starvation (S1B–S1D Fig), were treated with UV, α-MSH, or both under normal culture condition (Fig 1B–1D). The status of ciliogenesis was analyzed by immunofluorescence staining of the ciliary marker, Arl13b (Fig 1B) or ace-Tubulin (S1E Fig). While either UV or α-MSH slightly increased the number (Figs 1C and S1F) and length (Figs 1D and S1G) of cilia, UV and α-MSH co-treatment showed more obvious activity to trigger ciliogenesis (Figs 1B–1D and S1E–S1G). The UV/α-MSH-induced ciliogenesis can be mitigated by the MC1R antagonist agouti-signaling protein (ASIP) (Fig 1E–1G). Similar results were achieved in the human melanocyte cell line PIG1 (Fig 1H–1J) and the human melanoma cell line SK-MEL-2 (Figs 1K, 1L and S1H). As these cells have inferior ability in ciliogenesis, the ciliogenesis status was also tested in the background of cytochalasin D treatment, which increased the basal cilia level to magnify the ciliogenesis phenotype (Fig 1H–1L). Previous studies have demonstrated a ciliogenesis effect of UV through the dispersal of the centriolar satellite [22]. Collectively, these findings demonstrate that UV and α-MSH induce ciliogenesis in melanocytes and melanoma cells.

### MC1R signaling mediates the activity of α-MSH to stimulate ciliogenesis

**Fig 2:**
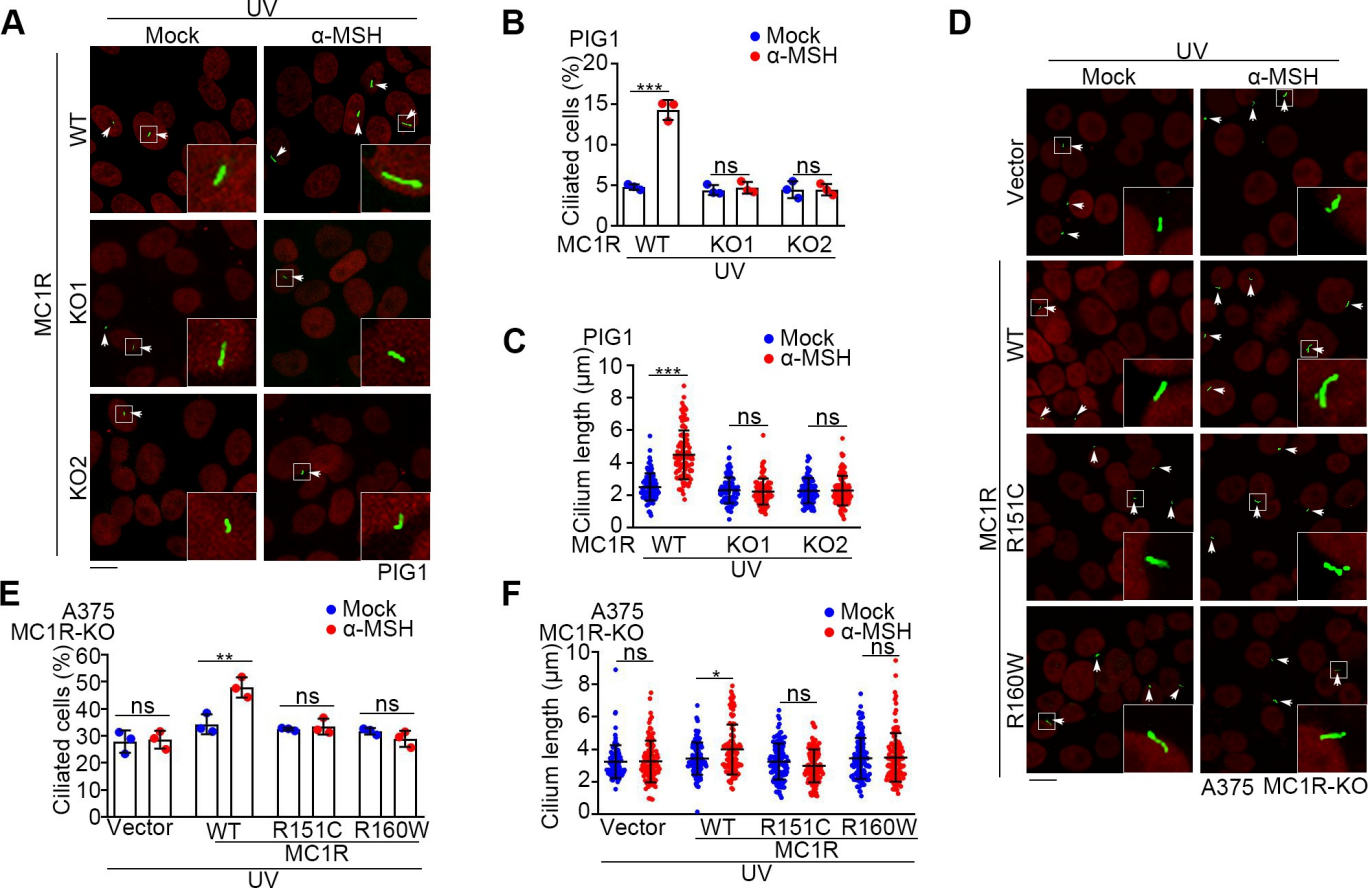
MC1R mediates the activity of α-MSH to trigger cilium formation. (A–C) Immunofluorescence images (A) and quantification of the percentage of ciliated cells (*n* = 3 independent experiments) (B) and cilium length (*n* ≥ 50 cells) (C) of PIG1 MC1R-WT and MC1R-KO cells that were mock-treated, or treated with 100 nM α-MSH for 24 h in the absence of serum after UV exposure. Cells were stained with the Arl13b antibody (green) and DAPI (red, pseudocolor). Ciliated cells were marked with white arrows. Scale bar, 10 μm. (D–F) Immunofluorescence images (D) and quantification of the percentage of ciliated cells (*n* = 3 independent experiments) (E) and cilium length (*n* ≥ 50 cells) (F) of A375 MC1R-KO rescued with the control vector or the MC1R variants. Cells were mock-treated or with 100 nM α-MSH for 24 h in the absence of serum after UV exposure and stained with the Arl13b antibody (green) and DAPI (red, pseudocolor). Ciliated cells were marked with white arrows. Scale bar, 10 μm. Data are presented as mean ± SD. Statistical significance was determined by unpaired two-tailed Student’s *t* test; *p < 0.05, **p < 0.01, ***p < 0.001; ns, not significant. See also S2 Fig. The underlying data for this figure can be found in S2 File and S1 Data. α-MSH, α-melanocyte-stimulating hormone; MC1R, melanocortin 1 receptor.

Given that α-MSH is the ligand for MC1R, we sought to investigate whether UV/α-MSH stimulates ciliogenesis through the MC1R signaling. To test this possibility, we used A375 human melanoma cell line, which contains an arginine-to-cysteine RHC variant at the residue 151 (R151C) of MC1R conferring its attenuated function [23]. We found that UV/α-MSH or ASIP failed to affect ciliogenesis in A375 cells (Figs 1M, 1N, and S1I–S1L). Next, we depleted MC1R in PIG1 cells by the CRISPR/Cas9 technology. Strikingly, UV/α-MSH promoted ciliogenesis in wild-type PIG1 (PIG1 MC1R-WT) cells, but not in MC1R knockout (PIG1 MC1R-KO) cells (Fig 2A–2C). To confirm the role of MC1R in ciliogenesis, we constructed A375 MC1R-KO cells and then rescued with WT MC1R or the RHC (R151C and R160W) variants (Figs 2D and S2A). We found that UV/α-MSH promoted ciliogenesis in A375 MC1R-WT cells, but not in the MC1R-KO, MC1R-R151C, or MC1R-R160W cells (Fig 2D–2F).

To explore the mechanism underlying UV/α-MSH-induced ciliogenesis, we compared the transcriptomes of A375 MC1R-WT and MC1R-KO cells treated with UV/α-MSH. Heatmap analysis of the significantly changed genes revealed that most ciliogenesis-promoting genes were up-regulated (S2B Fig). We selected several well-known ciliogenesis-related genes from the heatmap and set out for quantitative RT-PCR analysis or immunoblotting. Consistent with the transcriptomic results, several positive regulators of ciliogenesis, such as tau tubulin kinase 2 (TTBK2), intraflagellar transport 80 (IFT80), RAB3A interacting protein (RAB3IP), intraflagellar transport 74 (IFT74), PKHD1 ciliary IPT domain containing fibrocystin/polyductin (PKDH1), and Bardet–Biedl syndrome 2 (BBS2), were up-regulated in MC1R expressing cells, while the negative regulators of ciliogenesis, such as kinesin family member 24 (KIF24) and vesicle associated membrane protein 3 (VAMP3), were down-regulated (S2C and S2D Fig). UV treatment alone in A375 MC1R-WT cells did not change the expression of the related genes (S2E Fig). Thus, MC1R mediates the activity of UV/α-MSH for cilium formation, possibly through transcriptional regulation of genes critically involved in the ciliogenic process.

### Ciliary localization of MC1R is important for its regulation of melanogenesis

**Fig 3:**
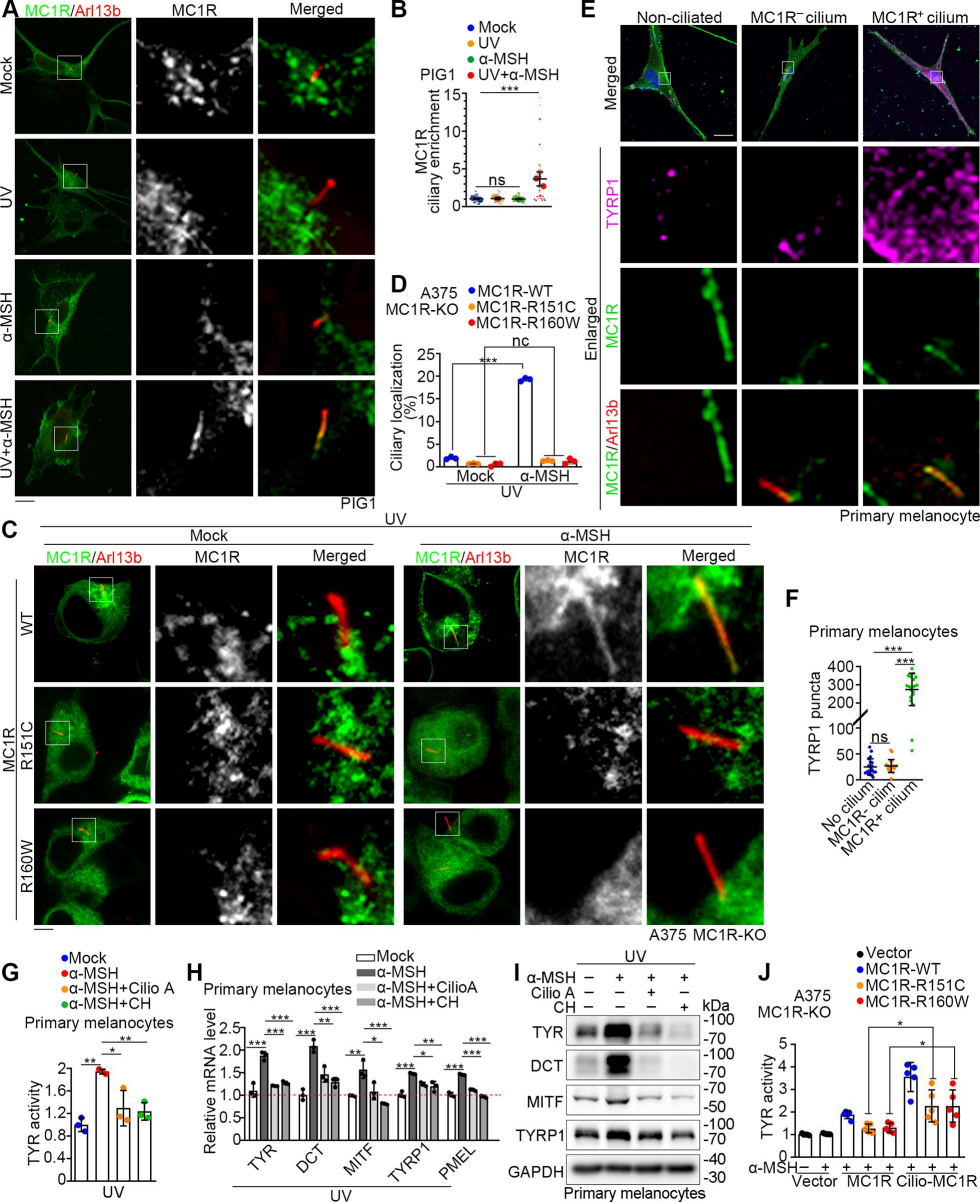
Ciliary localization of MC1R is critical for its role in melanogenesis. (A) Immunofluorescence images of PIG1 cells transfected with MC1R-mEmerald (green) and mock-treated, or treated with UV, 100 nM α-MSH, or both. Cells were treated with α-MSH for 36 h in the absence of serum after UV exposure. Cells were stained with the Arl13b antibody (red). Scale bar, 5 μm. (B) MC1R ciliary enrichment analysis of panel A as previously reported [16]. Ciliary and cell body intensity of MC1R was measured using ImageJ. Enrichment at the cilium is expressed as: (integrated density at the cilium)/(integrated density in the cell body). Enrichment > 1 indicates higher localization of mEmerald tagged MC1R at the primary cilium than at the cell body. Every replicate was represented as a superplot. (*n* = 30 ciliated cells from 3 different replicates) (C, D) Immunofluorescence images (C) of A375 MC1R-KO cells transfected with WT, R151C, or R160W MC1R-mEmerald (green) and mock-treated or treated with 100 nM α-MSH for 36 h in the absence of serum after UV exposure. Cells were stained with the Arl13b antibody (red). The percentage of ciliated cells with ciliary MC1R localization was quantified in panel D (*n* = 3 independent experiments). Scale bar, 5 μm. (E) Immunofluorescence images of MC1R-mEmerald transfected primary human melanocytes treated with UV/α-MSH (100 nM). Cells were treated with α-MSH for 36 h in the presence of serum after UV exposure. Cells were stained with the Arl13b (red) and TYRP1 (magenta) antibodies. Nuclei were stained with DAPI (blue). Representative images for non-ciliated cells, ciliated cells without ciliary MC1R (MC1R^−^ cilium), and ciliated cells with ciliary MC1R (MC1R^+^ cilium) were selected from the same slides. Scale bar, 10 μm. (F) Quantification of the number of TYRP1 puncta (*n* = 20 cells) as described in E. (G) Tyrosinase activity of primary human melanocytes that were mock treated, treated with 100 nM α-MSH, 100 nM α-MSH/30 μm ciliobrevin A (Cilio A), or 100 nM α-MSH/2 mM CH for 36 h in the absence of serum after UV exposure (*n* = 3 independent experiments). (H) Quantitative RT-PCR analysis of melanogenesis-related genes in primary human melanocytes treated as described in G (*n* = 3 independent experiments). TYR, tyrosinase; DCT, dopachrome tautomerase; MITF, melanocyte inducing transcription factor; TYRP1, tyrosinase related protein 1; PMEL, premelanosome protein. (I) Immunoblot analysis of melanogenesis-related proteins in primary human melanocytes treated as described in G. GAPDH served as a control. (J) Tyrosinase activity of A375 MC1R-KO cells rescued with different forms of MC1R (*n* = 5 independent experiments). Cilio-MC1R was constructed by fusing the ciliary protein Arl13b with WT, R151C, or R160W MC1R. Cells were treated with or without 100 nM α-MSH for 36 h in the absence of serum and UV. Data are presented as mean ± SD. Statistical significance was determined by one-way ANOVA; *p < 0.05, **p < 0.01, ***p < 0.001; ns, not significant. See also S3 Fig. The underlying data for this figure can be found in S1 Data. The uncropped blots are included in S1 Raw Images. α-MSH, α-melanocyte-stimulating hormone; CH, chloral hydrate; MC1R, melanocortin 1 receptor; UV, ultraviolet.

The melanocortin receptor family consists of 5 structurally and functionally related members [24], of which the ciliary localization of MC4R has been demonstrated to mediate its function in appetite and body weight control [11,13]. We found that MC1R-mEmerald localized to the cilium when PIG1 cells were treated with both UV and α-MSH, but not either of them alone (Fig 3A). Ciliary enrichment was analyzed according to the previous study [16], and the results showed that UV and α-MSH co-treatment increased the ciliary enrichment of MC1R (Fig 3B). To explore the feature of the ciliary localization of MC1R, we analyzed A375 MC1R-KO cells transfected with WT, R151C, or R160W MC1R-mEmerald (Fig 3C). Consistent with the observation in PIG1 cells (Fig 3A), WT MC1R entered the cilium upon UV/α-MSH stimulation, but the R151C and R160W variants were unable to enter the cilium (Fig 3C and 3D).

To characterize the ciliary function of MC1R in melanogenesis, we quantified the number of puncta formed by tyrosinase related protein 1 (TYRP1), a melanogenic transcriptional target of MC1R, in UV/α-MSH treated primary human melanocytes that were non-ciliated, ciliated without MC1R ciliary localization (MC1R^−^), or ciliated with MC1R ciliary localization (MC1R^+^) (Fig 3E and 3F). MC1R^+^ ciliated cells exhibited much more TYRP1 puncta than MC1R^−^ ciliated or non-ciliated cells. Then, we utilized ciliobrevin A and chloral hydrate to disrupt cilia in primary melanocytes (S3A and S3B Fig) and A375 MC1R-WT cells (S3C and S3D Fig). We found that disruption of cilia by these compounds suppressed the tyrosinase activity in primary melanocytes (Fig 3G). In the absence of cilia, the expression of melanogenesis-related proteins was also suppressed at both transcriptional (Fig 3H) and translational levels (Fig 3I). Similar results were obtained in A375 MC1R-WT cells (S3E–S3G Fig), indicating that the primary cilium is important for MC1R in regulating melanogenesis.

Next, we constructed an MC1R that is constitutively localized to the cilium (cilio-MC1R), by fusing the ciliary protein Arl13b with WT, R151C, or R160W MC1R (S3H and S3I Fig). These proteins showed robust ciliary localization even without UV/α-MSH treatment; the initially non-ciliary MC1R variants, R151C and R160W, also exhibited remarkable localization to the cilium upon fusion with Arl13b (S3I Fig). Though R151C and R160W MC1R had attenuated ability in inducing melanogenesis, the forced ciliary localization increased their ability to enhance the tyrosinase activity (Fig 3J). Thus, the ciliary localization of MC1R is critical for its activity to induce melanogenesis.

### The BBSome interacts with MC1R to promote its ciliary localization

Melanocortin receptors usually require associated proteins, such as melanocortin 2 receptor accessory proteins (MRAPs), for their proper localization, and the ciliary localization of MC4R is regulated by MRAP2 [16,25]. To test whether the ciliary localization of MC1R could be regulated by MRAPs, we co-expressed MRAP1 or MRAP2 in A375 MC1R-KO cells rescued with MC1R-mEmerald (A375 MC1R-mEmerald). We found that neither MRAP1 nor MRAP2 affected ciliary localization of MC1R (S4A–S4D Fig), which is consistent with the recent study [16].

**Fig 4:**
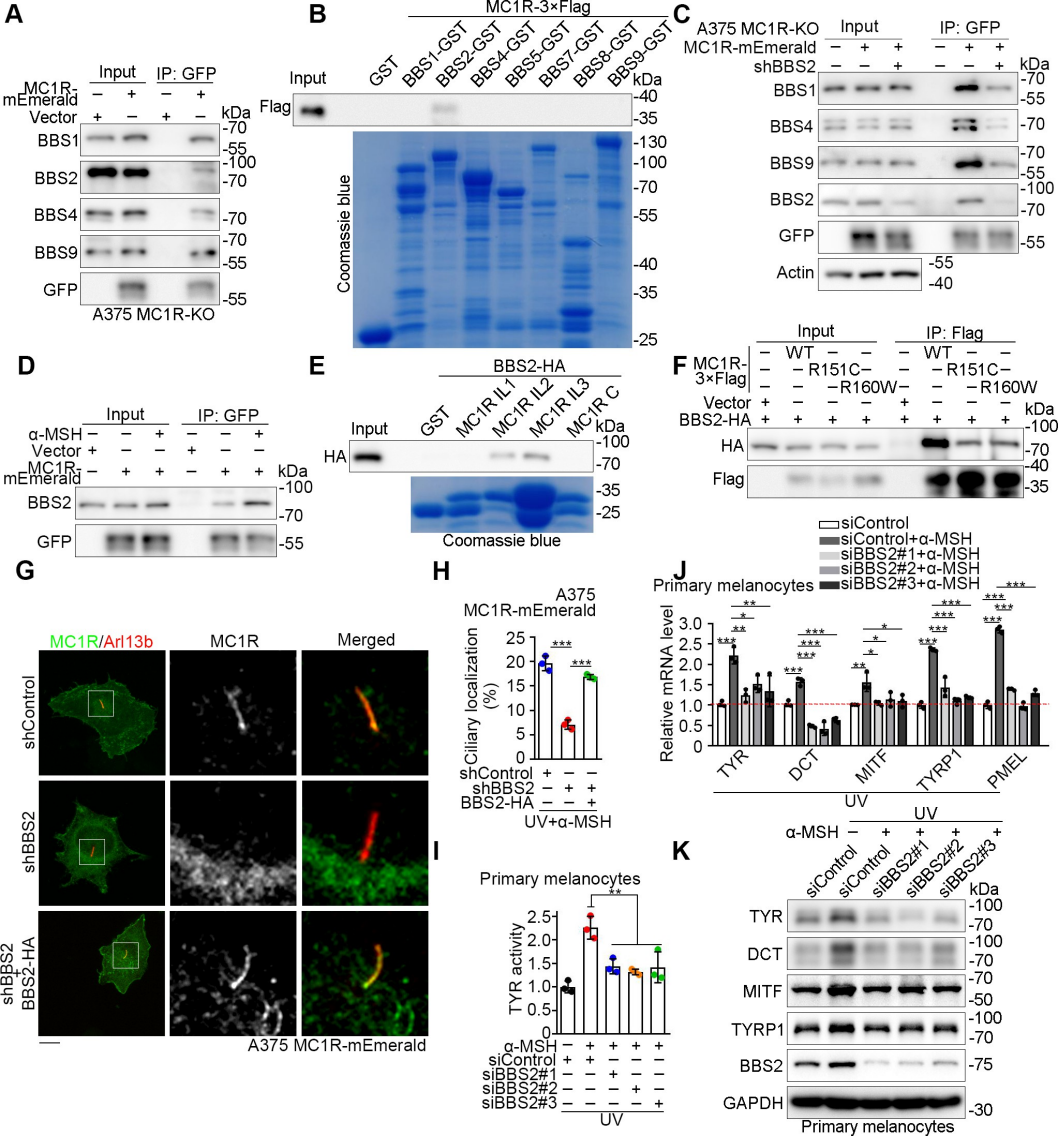
BBS2 interacts with MC1R to facilitate its ciliary localization. (A) Immunoprecipitation and immunoblotting showing the interaction between MC1R-mEmerald and endogenous BBSome proteins. A375 MC1R-KO cells were transfected with MC1R-mEmerald and treated with UV/α-MSH (100 nM). Cells were treated with α-MSH for 36 h in the absence of serum after UV exposure. (B) Pull-down analysis of the interaction between MC1R and BBSome proteins. (C) Immunoprecipitation and immunoblotting showing the interaction between MC1R-mEmerald and BBSome proteins under the BBS2 knockdown condition. A375 MC1R-KO cells were transfected with or without BBS2 shRNAs and treated with UV/α-MSH (100 nM). Cells were treated with α-MSH for 36 h in the absence of serum after UV exposure. (D) Immunoprecipitation and immunoblotting showing the interaction between MC1R-mEmerald and endogenous BBS2 under the UV radiation condition. A375 MC1R-KO cells were transfected with MC1R-mEmerald and treated with or without 100 nM α-MSH for 36 h in the absence of serum after UV exposure. (E) Pull-down analysis of the interaction between truncated MC1Rs and BBS2. IL, intracellular loop; C, C-terminal. (F) Immunoprecipitation and immunoblotting showing the interaction of MC1R variants with BBS2. HEK293T cells were transfected with the variants of MC1R-3×Flag and BBS2-HA and immunoprecipitated with the Flag antibody. (G, H) Immunofluorescence images (G) of A375 MC1R-KO cells rescued with MC1R-mEmerald (A375 MC1R-mEmerald, green) and transfected with control or BBS2 shRNAs in the presence or absence BBS2-HA. Cells were treated with α-MSH (100 nM) for 36 h in the absence of serum after UV exposure and were stained with the Arl13b antibody (red). The percentage of ciliated cells with ciliary localization of MC1R was quantified in panel H (*n* = 3 independent experiments). Scale bar, 5 μm. (I) Tyrosinase activity of primary human melanocytes transfected with control or BBS2 siRNAs and treated with or without 100 nM α-MSH for 36 h in the absence of serum after UV exposure (*n* = 3 independent experiments). (J) Quantitative RT-PCR analysis of melanogenesis-related genes of primary human melanocytes transfected with control or BBS2 siRNAs. Cells were treated with or without 100 nM α-MSH for 36 h in the absence of serum after UV exposure (*n* = 3 independent experiments). (K) Immunoblot analysis of melanogenesis-related proteins of primary human melanocytes transfected with control or BBS2 siRNAs. Cells were treated with or without 100 nM α-MSH for 36 h in the absence of serum after UV exposure. GAPDH served as a control. Data are presented as mean ± SD. Statistical significance was determined by one-way ANOVA; *p < 0.05, **p < 0.01, ***p < 0.001. See also S4 Fig. The underlying data for this figure can be found in S1 Data. The uncropped gels and blots are included in S1 Raw Images. α-MSH, α-melanocyte-stimulating hormone; BBS, Bardet–Biedl syndrome; MC1R, melanocortin 1 receptor; UV, ultraviolet.

Ciliary membrane proteins are transported across the transition zone to enter the cilium, which is mediated by IFT particles [26]. Adaptor proteins linking IFTs and membrane proteins, including Tulp3 and BBSome complex, are speculated to facilitate the ciliary localization of GPCRs [14,15]. We found that the ciliary localization of MC1R was increased by Tulp3 co-expression (S4E and S4F Fig). However, the endogenous expression of Tulp3 is weak in melanocyte-derived cell lines (S4G Fig), indicating the existence of other mechanisms in regulating the ciliary localization of MC1R. To test whether the BBSome is involved in the ciliary trafficking of MC1R, we employed co-immunoprecipitation assays. We found that MC1R-mEmerald interacted with several members of the BBSome in A375 MC1R-KO cells (Fig 4A). In addition, MC1R-3×Flag interacted with HA-tagged BBSome proteins (S4H Fig). Furthermore, MC1R-3×Flag was pulled down by purified GST-BBS2, but not other BBSome proteins (Fig 4B). Knockdown of BBS2 weakened the interaction between MC1R and the BBSome (Fig 4C), indicating that the BBSome interacts with MC1R through BBS2. We also found that the interaction between BBS2 and MC1R was increased by α-MSH stimulation (Fig 4D).

The BBSome has been reported previously to bind the third loop or the C-terminus of several GPCRs [27–29]. We purified the intracellular loops and the C-terminus of MC1R (S4I Fig) and tested their interaction with BBS2. We found that BBS2 was pulled down by the second intracellular loop and the third intracellular loop of MC1R (Fig 4E). Interestingly, the second loop of MC1R contains residues associated with the R151C and R160W RHC variants (S4I Fig). Co-immunoprecipitation showed that the R151C and R160W variants had an attenuated ability to interact with BBS2 (Fig 4F). Considering that the R151C and R160W variants were unable to locate to the cilium, these findings suggest that the BBSome may play a role in the ciliary localization of MC1R.

To examine whether MC1R locates to the cilium through interacting with the BBSome, we depleted the expression of BBS2 in melanocytes. We found that knockdown of BBS2 ablated the ciliary localization of MC1R, which was rescued by expression of exogenous BBS2-HA (Figs 4G, 4H, and S4J). Disrupting BBSome formation by depleting other BBSome components BBS1 or BBS5 also inhibited the ciliary localization of MC1R (S4K–S4M Fig). In addition, knockdown of BBS2 in primary melanocytes suppressed the tyrosinase activity (Fig 4I), the transcription of melanogenesis-related genes (Fig 4J), and the expression of melanogenesis-related proteins (Fig 4K). Taken together, these results indicate that the BBSome mediates the ciliary localization of MC1R to promote melanogenesis.

### Palmitoylation of MC1R stimulates its ciliary localization

**Fig 5:**
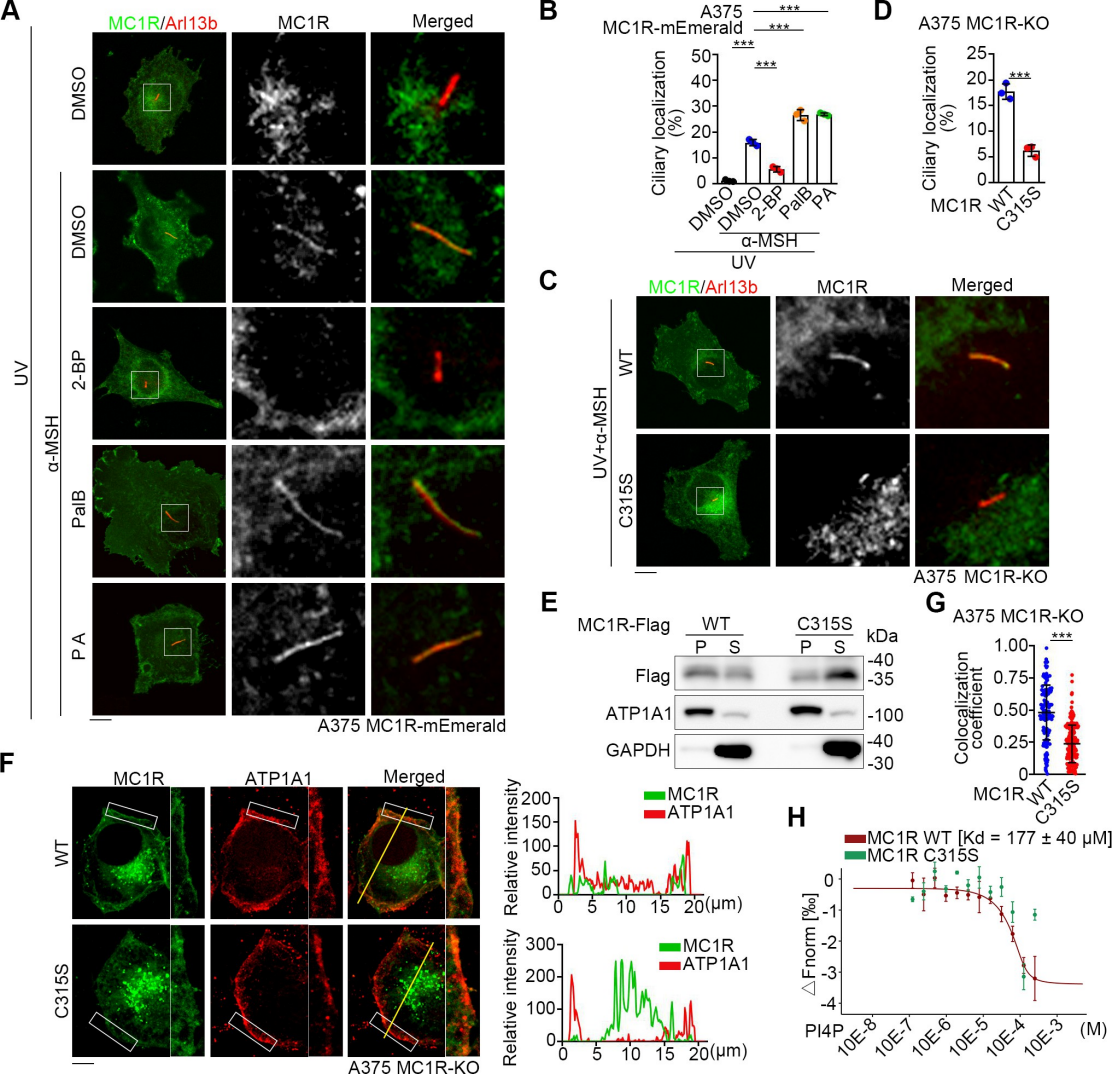
MC1R palmitoylation at C315 promotes its ciliary localization. (A, B) Immunofluorescence images (A) of A375 MC1R-KO cells rescued with MC1R-mEmerald (green) and treated with vehicle (DMSO), 100 nM α-MSH, 100 nM α-MSH/25 μm 2-bromopalmitate (2-BP), 100 nM α-MSH/1 μm palmostatin B (PalB), or 100 nM α-MSH/100 μm PA for 36 h in the absence of serum after UV exposure. Cells were stained with the Arl13b antibody (red). The percentage of ciliated cells with ciliary localization of MC1R was quantified in panel B (*n* = 3 independent experiments). Scale bar, 5 μm. (C, D) Immunofluorescence images (C) of A375 MC1R-KO cells rescued with WT and C315S MC1R-mEmerald (green) and treated with UV/α-MSH (100 nM). Cells were treated with α-MSH for 36 h in the absence of serum after UV exposure. Cells were stained with the Arl13b antibody (red). The percentage of ciliated cells with ciliary localization of MC1R was quantified in panel D (*n* = 3 independent experiments). Scale bar, 5 μm. (E) Immunoblot analysis of MC1R in different fractions of HEK293T cells transfected with WT or C315S MC1R-3×Flag. P, pellet fraction (membrane fraction); S, soluble fraction. (F) Immunofluorescence images (left) and line scan (right) of A375 MC1R-KO cells rescued with WT and C315S MC1R-mEmerald (green) and stained with the ATP1A1 antibody (red). Scale bar, 5 μm. (G) Pearson’s colocalization analysis of MC1R and ATP1A1 for images shown in panel F. *n* ≥ 50 cells. (H) Binding affinity of WT and C315S MC1R to PI4P measured by the MST assay. Data are presented as mean ± SD. Statistical significance was determined by one-way ANOVA (B) or unpaired two-tailed Student’s *t* test (D and G); ***p < 0.001. See also S5 Fig. The underlying data for this figure can be found in S1 Data. The uncropped blots are included in S1 Raw Images. MC1R, melanocortin 1 receptor; PA, palmitic acid; UV, ultraviolet.

MC1R has been reported to undergo palmitoylation at cysteine 315 (C315) upon UV stimulation and this modification is important for its activity in melanogenesis and melanomagenesis [20,21]. To examine whether the palmitoylation of MC1R affects its ciliary localization, we treated cells with compounds inhibiting palmitoylation (2-bromopalmitate) or promoting palmitoylation (palmostatin B and palmitic acid) (Figs 5A and S5A). We found that UV/α-MSH-induced ciliary localization of MC1R was inhibited by 2-bromopalmitate and enhanced by palmostatin B and palmitic acid (Fig 5A and 5B). Furthermore, the palmitoylation-deficient mutant of MC1R, C315S, failed to locate to the cilium (Fig 5C and 5D). Therefore, palmitoylation is required for the ciliary localization of MC1R.

Palmitoylation is known to affect the localization of membrane proteins by modulating their interaction with other proteins or by stabilizing their membrane association [30]. However, the C315S mutation did not affect the interaction of MC1R with BBS2 (S5B Fig). In contrast, the C315S mutation reduced the enrichment of MC1R in membrane fractions in cellular fractionation analysis (Fig 5E). Immunofluorescence microscopy showed that C315S MC1R displayed reduced localization at the plasma membrane (Fig 5F and 5G). Microscale thermophoresis (MST) assays further revealed that C315S MC1R had a reduced affinity to phosphatidylinositol 4-phosphate (PI4P), a marker of the ciliary membrane [31,32], as compared to WT MC1R (Fig 5H). These results suggest that the palmitoylation of MC1R facilitates its ciliary membrane association but is dispensable for its interaction with the BBSome.

### Ciliary MC1R triggers a sustained cAMP signaling to induce melanogenesis

**Fig 6:**
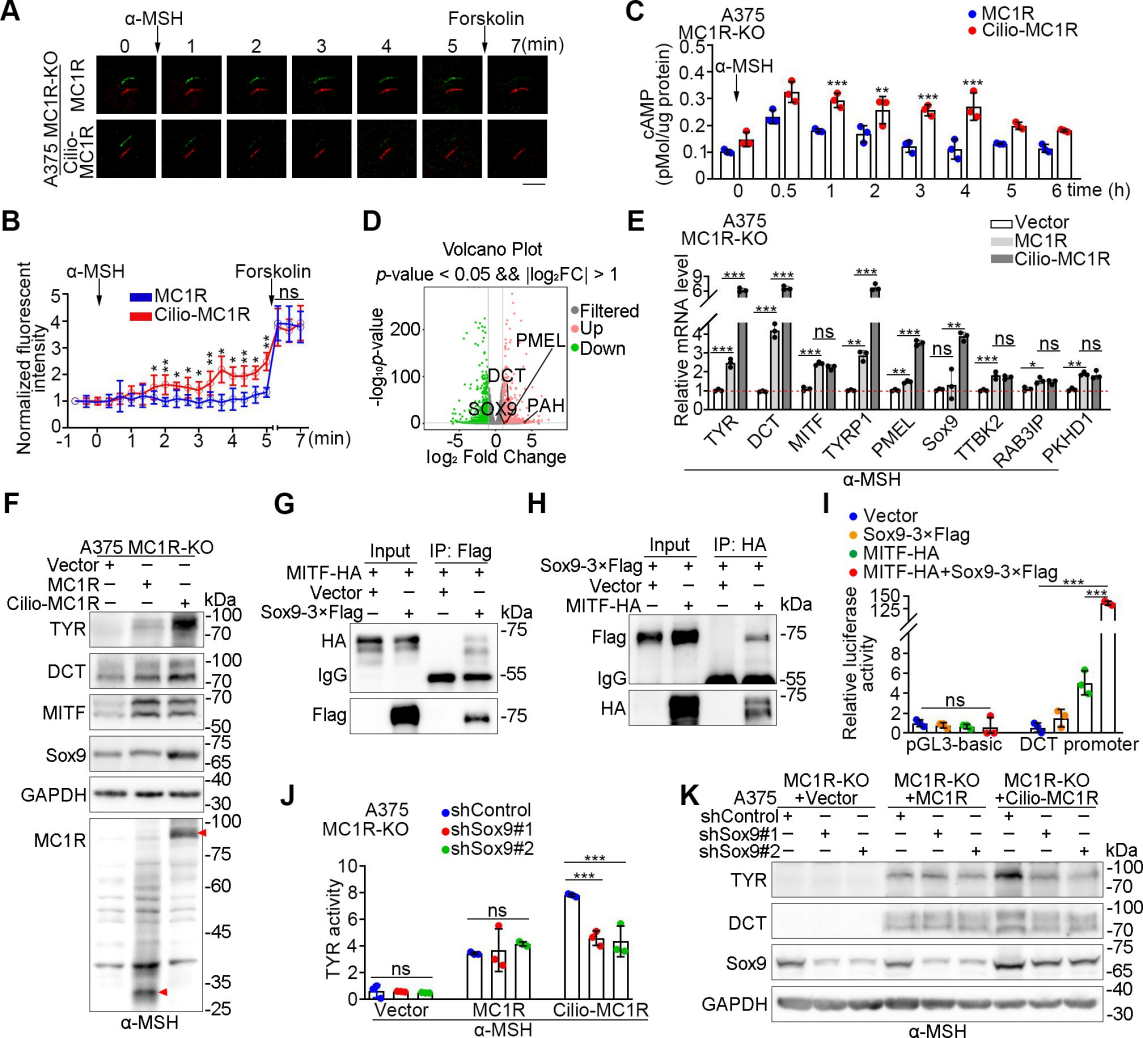
Cilio-MC1R induces a sustained cAMP signaling during skin pigmentation. (A, B) Detection of ciliary cAMP levels by cilia-targeted cADDis (green) and the reference ciliary marker 5HT6-mCherry (red) in A375 MC1R-KO cells rescued with MC1R or cilio-MC1R (constructed by fusing the ciliary protein Arl13b with MC1R). Cells were pre-serum-starved for 24 h and cultured in the absence UV. Panel A shows time-lapse images of cADDis (green) and cilia (red) after the addition of 1 μm α-MSH (denoted by arrow). Then, 100 μm forskolin was added at 6 min (denoted by arrow). Scale bar, 2.5 μm. In panel B, the fluorescence intensities were normalized to the time point of −1 min (*n* = 5 ciliated cells from 5 independent experiments). The normalized fluorescence intensity is expressed as: (the fluorescence intensity of mCherry / the fluorescence intensity of cADDis) / (the fluorescence intensity of mCherry at −1 min / the fluorescence intensity of cADDis at −1 min). (C) Time course of total cellular cAMP levels of A375 MC1R-KO cells rescued with MC1R or cilio-MC1R upon stimulation with 1 μm α-MSH. Cells were pre-serum-starved for 24 h and cultured in the absence UV. (D) Volcano plots of differentially expressed genes in A375 MC1R-KO cells rescued with WT MC1R or cilio-MC1R. Cells were cultured in the absence of serum and UV, and treated with 100 nM α-MSH for 12 h before mRNA extraction. Genes with fold change >2 and adjusted *p*-value <0.05 were considered as differentially expressed. Melanogenesis-related genes were highlighted and marked on the spot. (E) Quantitative RT-PCR analysis of the indicated genes of A375 MC1R-KO cells transfected with the control vector, MC1R, or cilio-MC1R. Cell were treated with 100 nM α-MSH for 12 h before mRNA extraction (*n* = 3 independent experiments). Cells were cultured in the absence of serum and UV. (F) Immunoblot analysis of the indicated proteins in A375 MC1R-KO cells rescued with the control vector, MC1R, or cilio-MC1R. Cells were treated with 100 nM α-MSH for 24 h in the absence of serum and UV before lysis. GAPDH served as a control. (G, H) Immunoprecipitation and immunoblotting showing the interaction between Sox-3×Flag and MITF-HA. HEK293T cells were transfected with the indicated plasmids and immunoprecipitated with antibodies against Flag (G) and HA (H). (I) Dual-luciferase reporter assay of the pGL3-basic or *DCT* promoter transfected with the indicated plasmids. The relative luciferase activity was calculated as the luciferase activity normalized with the control vector (*n* = 3 independent experiments). (J) Tyrosinase activity of A375 MC1R-KO cells rescued with the control vector, MC1R, or cilio-MC1R. Cells were transfected with control or Sox9 shRNAs and treated with 100 nM α-MSH for 24 h in the absence of serum and UV before detection (*n* = 3 independent experiments). (K) Immunoblot analysis of the indicated proteins in A375 MC1R-KO cells rescued with the control vector, MC1R, or cilio-MC1R. Cells were transfected with control or Sox9 shRNAs and treated with α-MSH for 24 h in the absence of serum and UV before lysis. GAPDH served as a control. Data are presented as mean ± SD. Statistical significance was determined by unpaired two-tailed Student’s *t* test (B and C) or one-way ANOVA (E, I, and J); *p < 0.05, **p < 0.01, ***p < 0.001; ns, not significant. See also S6 Fig. The underlying data for this figure can be found in S1 Data. The uncropped blots are included in S1 Raw Images. α-MSH, α-melanocyte-stimulating hormone; MC1R, melanocortin 1 receptor; UV, ultraviolet.

To investigate the function of ciliary MC1R in the cAMP-dependent signaling, we used cADDis, a genetically encoded fluorescent sensor that specifically detects ciliary cAMPs [33]. Increased ciliary cAMPs reduce ciliary green fluorescence signal of the sensor, whose intensity can be normalized to that of ciliary marker for comparison [33]. By using the cADDis sensor, we found that ciliary cAMPs were significantly increased by α-MSH stimulation in A375 MC1R-KO cells expressing cilio-MC1R, but not WT MC1R (Fig 6A and 6B). The ciliary elevation of cAMP by cilio-MC1R was further confirmed by the ratiometric cAMP reporter cAMPinG1 (S6A–S6C Fig) [34]. When total cellular cAMP levels were measured, we observed that cilio-MC1R induced a sustained cAMP signaling over time (Fig 6C).

The primary cilium is known to distinguish different subcellular pools of cAMP to convey distinct information [9,10]. Transcriptomic analysis of A375 MC1R-KO cells rescued with cilio-MC1R versus WT MC1R showed that several melanogenesis-related genes were up-regulated in the cilio-MC1R group (Fig 6D). Quantitative RT-PCR confirmed the up-regulation of melanogenesis-related genes, but not genes irrelevant to melanogenesis (Fig 6E). Although the mRNA and protein levels of MITF were not obviously altered, those of SRY-box transcription factor 9 (Sox9), a transcription co-factor for MITF, were significantly up-regulated by cilio-MC1R (Fig 6E and 6F). To identify the pool of MC1R for ciliogenesis, we constructed a ciliary localization-deficient MC1R by fusing MC1R with Arl13b V359A, a missense mutant of Arl13b that prevents its ciliary localization (S6D Fig) [10,35]. We found that the ciliary localization-deficient MC1R could still promote the expression of ciliogenesis-related genes (S6E Fig), indicating that the ciliogenesis function of MC1R is not cilium-specific and can be conducted by the plasma membrane pool of MC1R.

Sox9 is known to enhance the transcription ability of MITF to increase the expression of MITF-targeted proteins, and to stimulate skin pigmentation [36,37]. Consistent with previous findings [36], we found that Sox9 interacted with MITF (Fig 6G and 6H). To test the transcriptional activity of MITF, dual-luciferase reporter assays were applied using the promoter of *dopachrome tautomerase* (*DCT*). The combination of Sox9 and MITF caused a more extensive activation of the *DCT* promoter, indicating a synergistic effect between Sox9 and MITF (Fig 6I). In addition, knockdown of Sox9 significantly inhibited the melanogenesis induced by cilio-MC1R (Fig 6J and 6K). Taken together, these results suggest that cilio-MC1R induces a sustained cAMP signaling and up-regulates the expression of melanogenesis-related proteins, such as Sox9, to simulate the pigmentation process.

## Discussion

Melanocortin receptors consist of 5 members, which are structurally conserved and play important roles in different tissues [38]. It has been reported that MC4R was localized in the cilium of a subset of hypothalamic neurons, and obesity-associated MC4R mutations impaired its ciliary localization [11]. Further study demonstrated that cilia were required specifically in MC4R-expressing neurons for the control of energy homeostasis [13]. Recently, Bernard and colleagues [16] showed that the ciliary localization of MC4R can be regulated by MRAP2, revealing the requirement for specific accessory proteins in the ciliary localization of GPCRs. The ciliary regulation of other melanocortin receptor members remains to be further explored.

The MC1R signaling plays a critical role in melanogenesis, but the molecular details and regulatory mechanisms remain unclear. In the present study, our data show that UV and α-MSH promote the formation of primary cilia through MC1R-mediated transcriptional regulation of ciliogenesis-related genes. MC1R then enters the cilium in a palmitoylation-dependent manner, thereby leading to a sustained melanogenesis. The transport of ciliary proteins is known to rely on the coordination between IFT proteins and adaptors, such as the BBSome [8]. The BBSome recognizes specific ciliary localization sequences (normally located in the third intercellular loop or the C terminal of GPCR proteins) in ciliary membrane proteins and regulates their entry or exit of cilia [27,29]. In addition, the BBSome mediates melanosome transport by regulating organelle trafficking [39], indicating a potential role for the BBSome in melanosome regulation. In this study, we demonstrate that the BBSome interacts with MC1R to regulate its ciliary localization and function in melanogenesis. Our findings thus demonstrate a previously undefined role for the primary cilium in MC1R-mediated melanogenesis.

The RHC variants of MC1R are notorious not only for their reduced ability in melanogenesis, but also for their enhanced association with melanomagenesis [17]. The MC1R variant alleles (D84E, R151C, R160W, and D294H) strongly associated with the RHC phenotype have been designated as “*R*” alleles, whereas the variant alleles (V60L, V92M, and R163Q) that have a relatively weak association with RHC are known as “*r*” alleles. It should be noted that the melanomagenesis tendency of the RHC variants are partially, but not totally, attributable to their vulnerable pigmentation [40–42]. The RHC variants behave differently from WT MC1R and induce a weakened cAMP signaling, due to differences in their associated proteins [43], posttranslational modifications [20,21], or surface ligand binding [44]. Further studies are warranted to elucidate the mechanisms underlying the reduced melanogenesis of the RHC variants.

Our study reveals that the second intercellular loop of MC1R, which contains 2 common RHC variant alleles (R151C and R160W), is responsible for its interaction with the BBSome. The R151C and R160W variants have an attenuated capacity in BBSome binding and thus a reduced ability in ciliary localization. On the other hand, these RHC variants are less palmitoylated in response to UV/α-MSH stimulation [21] and palmitoylation regulates the ciliary localization of MC1R, it is not difficult to understand why the R151C and R160W variants fail to locate to the cilium. In fact, the RHC variants are not totally loss-of-function receptors and retain considerable capacity in mediating the cAMP signaling [45]. We demonstrate that enforced ciliary localization of the RHC variants elevates the melanogenesis capacity of these proteins. Thus, the attenuated ciliary localization may underlie the compromised functions of R151C and R160W variants in stimulating cAMP production.

The role for the cilium in regulating the melanocyte behavior has been implied that ciliogenesis is greatly attenuated in melanoma compared with associated conventional melanocytic nevi [4]. A previous study showed that cilium induction inhibited melanin synthesis in melanocytes probably by hedgehog signaling, while the detailed mechanisms remain mysterious [46]. However, in retinal pigment epithelial cells derived from pluripotent stem cells of ciliopathy patients, cilia defects are associated with the malformation of melanosome [47]. Thus, the role of cilia and the underlying mechanisms in melanogenesis deserve further exploration. Our results that ciliary MC1R-enhanced melanogenesis suggested a positive regulatory role for cilia in pigmentation. Considering the experimental differences (such as with or without UV treatment), it is possible that the cilium is a two-edged sword in pigmentary regulation by stimulating various signalings in different conditions. It remains to be explored to what extent melanocytes are ciliated in vivo. The ciliogenesis functions of MC1R may be a security mechanism to replenish sufficient ciliated cells when ciliation rates are low. It may also stabilize pre-formed cilia to ensure the proper process of MC1R ciliary signaling.

The length of the primary cilium has been reported to be increased by cAMP in epithelial and endothelial cells [48,49]. However, there is also evidence that cAMP elevation promotes cilium resorption [50]. The dual effect of cAMP on primary cilia may be attributed to the culture conditions, such as the presence or absence of serum [50]. Nanobody-directed cAMP local activation reveals that cilium length is increased by cAMP synthesized in the cilium, while decreased by cAMP synthesized in the cell body [51], pointing to a hypothesis in functional distinctions of the cAMP signaling compartmentalization. In addition, it is reported that cilium elongation can be promoted by some Gαs-coupled GPCRs, such as E-type prostanoid receptor 4 (EP4) [52] and 5-hydroxytryptamine receptor 6 (5HT6) [53], and be shortened by Gαi-coupled dopamine receptor type 2 (D2) [54] and melanin-concentrating hormone receptor (MCHR1) [55]. Interestingly, cilium elongation has also been observed upon overexpression of the Gαi-coupled SSTR3 [56], and some studies report that mutations affecting 5HT6 functions have little to no impact on preventing the cilium elongation effect [56,57]. Considering all these contradictions, the cilium regulation mechanisms by GPCRs are complicated and may involve a balance of different signaling pathways.

GPCRs usually undergo rapid desensitization and internalization upon stimulation [58]. However, a sustained G protein signaling can take place within compartments otherwise than the plasma membrane, such as the internalized endosomes [59,60]. These compartments are responsible for the late phase activation of the GPCR and a sustained cAMP signaling. The primary cilium is an ideal signaling hub due to its enlarged surface area and robust capacity for cAMP collection [33,61]. Ciliary cAMPs have recently been shown to transmit information in a pattern different from extraciliary cAMPs [9,10]. In this scenario, it is easy to understand why ciliary GPCRs exert a function distinct from the plasma membrane-localized GPCRs [62]. It is demonstrated that ciliary cAMP signalosome promotes a specific gene expression program different from the cell body, as distinct geometries of the cilium and cell body differentially activate local effectors, such as PKA and CREB [9,10]. The transcription factor CREB shuttles through the cilium, where it is phosphorylated by the ciliary PKA, to regulate gene expression [9]. In our study, ciliary MC1R specifically induces the expression of Sox9 to potentiate the transcriptional activity of MITF, which may explain the enhanced melanogenesis by ciliary MC1R. It remains to be determined whether and how the ciliary function of MC1R is conducted by ciliary-specific effectors. In contrast, the role of MC1R in mediating ciliogenesis seems to be independent of its ciliary localization, as its ciliary exclusion did not affect the induction of ciliogenesis-related genes (S6D and S6E Fig) and their expressions are indiscriminate between the cilio-MC1R and WT MC1R groups (Fig 6E). Thus, the ciliogenesis function of MC1R is not cilium specific.

In the present study, we show that ciliary MC1R mediates a more sustained cAMP signaling (Fig 6C) than the plasma membrane-localized MC1R. In addition, ciliary MC1R induces the expression of melanogenesis-related genes in a more intense manner (Fig 6E and 6F). The robust ciliary effect of MC1R (Figs 3E, 3F, and 6I) may explain the increase of overall melanogenesis by the ciliary MC1R despite a low ciliary targeting rate. A higher concentration of ciliary cAMP and PKA activity than cytosolic has been reported [33]. The ciliary cAMP compartment from the cytoplasm can be maintained by ciliary-enriched cAMP signaling cascade components, such as PDE4 [9]. However, there are studies that debate an elevated basal cAMP in the cilium [61]. The differences in cAMP level detection between the 2 compartments may depend on the properties of the cAMP reporters used. Besides, cAMP can diffuse freely through the transition zone, so it is unsurprising that its level ultimately equilibrates in the cilium and cytosol [10]. It is also showed that cAMP can diffuse from the cytosol to the cilium, but cytosolic cAMP does not match the levels of local cAMP production in this organelle [10]. Considering that PKA activation requires a rather high cAMP concentration threshold in vivo [63], the greater surface to volume ratio of the cilium endows it a more efficient activation of the ciliary cAMP effectors [10]. Other explanations for the prolonged ciliary MC1R activity may include the distinct lipid composition, high intraciliary Ca^2+^, ciliary-specific enrichment of cAMP cascade components, and GPCR regulatory proteins, which merit further exploration. Overall, our findings provide novel insights into the molecular mechanisms regulating melanogenesis and skin pigmentation.

## Materials and methods

### Plasmids, siRNAs, and shRNAs

Mammalian expression plasmids for MC1R-3×Flag, MC1R-mEmerald, and BBS2-HA were generated by insertion of the cDNAs into the pLVX-IRES puro vector (Addgene), and their mutants were generated by PCR and site-directed mutagenesis. BBS2 siRNAs (#1: 5′- CUCGGUCAGAACUUUCUGUUA-3′; #2: 5′-CGGGAUGCAAUUCGAAGCAAU-3′; #3: 5′-CCUUGGUGAGUUGUACAAAUU-3′) were synthesized by RiboBio (Guangzhou, China). shRNAs for BBS2 or Sox9 were cloned into the pLKO.1 vector (Addgene).

### Antibodies and chemicals

Primary antibodies used in this study included the following: β-actin (Abways, AB0035; 1:1,000), ace-tubulin (Sigma-Aldrich, T7451; 1:1,000), ATP1A1 (Proteintech, 14418-1-AP; 1:1,000), Arl13b (Proteintech, 17711-1-AP; 1:2,000), BBS1 (Proteintech, 21118-1-AP; 1:1,000), BBS2 (Proteintech, 11188-2-AP; 1:1,000), BBS4 (Proteintech, 12766-1-AP; 1:1,000), BBS9 (Proteintech, 14460-1-AP; 1:1,000), Flag (Sigma-Aldrich, F1804; 1:2,000; Proteintech, 20543-1-AP; 1:1,000), DCT (Santa Cruz Biotechnology, sc-74439; 1:1,000), GFP (Abways, AB0045; 1:1,000; Abways, AB0005; 1:1,000), HA (Sigma-Aldrich, H9658; 1:5,000), MC1R (Signalway Antibody, 36969; 1:500), MITF (Proteintech, 13092-1-AP; 1:1,000; Santa Cruz Biotechnology, sc-515925; 1:1,000), TYR (Santa Cruz Biotechnology, sc-20035; 1:1,000), TYRP1 (Santa Cruz Biotechnology, sc-166857; 1:1,000). Secondary antibodies included Alexa Fluor 488 or Alexa Fluor 568 conjugated-anti-mouse or anti-rabbit antibodies (Invitrogen) for immunofluorescence microscopy and horseradish peroxidase-conjugated goat anti-mouse or anti-rabbit antibodies (Jackson ImmunoResearch Laboratories) for immunoblotting. α-MSH, cytochalosin D, and ciliobrevin A were purchased from Sigma-Aldrich. 2-Bromopalmitate, palmostatin B, and palmitic acid were from Santa Cruz Biotechnology. ASIP was from Cusabio Technology.

### Cell culture, transfection, and UV radiation

HEK293T, PIG1, A375, and SK-MEL-2 cells were cultured in the DMEM supplemented with 10% fetal bovine serum at 37°C in a humidified atmosphere containing 5% CO_2_. Primary human melanocytes were isolated from normal discarded foreskins as described previously [21] and were cultured in RPMI1640 (Biological Industries) supplemented with 200 nM phorbol 12-myristate 13-acetate (Sigma-Aldrich), 100 pM cholera toxin (Sigma-Aldrich), 10 nM endothelin 1 (MedChemExpress), and 10 ng/ml recombinant human stem cell factor (Sangon Biotech). The protocol to obtain primary human melanocytes from healthy donors was approved by the Ethics Committee of The 2nd Hospital, Zhejiang University (2022–0732). Plasmids were transfected into cells with polyethylenimine (Polyscience) or Lipofectamine 3000 (Thermo Fisher Scientific), and siRNAs were transfected with Lipofectamine RNAiMAX (Invitrogen).

For UV radiation, cells were washed with PBS and exposed to UVB light bulbs (Philips) through a small volume of PBS at a dose of 100 J/m^−2^ (2.5 W/m^2^, 40 s). UV-exposed cells were then cultured and subjected to normal culture medium, serum starvation, or drug treatments for the indicated times in the figure legends until the time of the assay.

### Generation of stable cell lines

To generate MC1R knockout cell lines, 2 target oligos (5′-CCCACAGCCATCCCCCAGCT-3′ and 5′-AGCCAGCCCCAGCTGGGGGA-3′) ligated into the gRNA vector were transfected into PIG1 or A375 cells with a plasmid expressing the Cas9 protein [64,65]. After transfection for 24 h, the cells were monocloned into 96-well plates. Successful knockout clones were confirmed by sequencing.

To generate cells with stable expression of MC1R variants, A375 MC1R-KO cells were rescued with MC1R variants, cilio-MC1R, or MC1R-mEmerald. Plasmids were co-transfected with the psPAX2 and pMD2.G plasmids into HEK293T cells. Lentiviruses were collected 48 h after transfection, and then used to infect cells for 24 h in the presence of 10 μg/ml polybrene (Sigma-Aldrich). The infected cells were selected with 2 μg/ml puromycin (Sigma-Aldrich).

### Immunofluorescence microscopy

Cells were fixed with 4% paraformaldehyde in PBS at 37°C for 15 min followed by permeabilization in 0.15% Triton X-100 and washing with PBS. After blocking in 3% BSA, the samples were incubated with primary antibodies and then with Alexa Fluor-conjugated secondary antibodies. The specimens were examined under an SP8 confocal microscope (Leica) equipped with a 63×/1.40 numerical aperture objective lens and analyzed using the analysis tools of the Leica Application Suite X software and the ImageJ software (National Institutes of Health). The pinhole was set to 1 airy unit at maximal optical resolution before gain and offset were calibrated, and 405 nm (emission detected between 420 and 480 nm), 488 nm (emission detected between 500 and 550 nm), 568 nm (emission detected between 580 and 630 nm), or 647 nm (emission detected between 655 and 700 nm) laser lines were used. Images were acquired with the PMT detector.

Quantification of ciliary lengths was calculated with at least 50 cilia from 3 independent experiments. Images were scrolled down to the bottom of the cells to set the bottom, and then scrolled up to the top of the cells to set the top. Z-stacks (0.5 μm step) were collected and stacked images were converted to maximum fluorescence intensity projection. At least 3 randomly chosen fields of view were selected per condition per experiment, and all cilia within the field were manually measured using the line segment tool in ImageJ in double-blind manner.

### Immunoblotting and immunoprecipitation

For immunoblotting, protein samples were subjected to SDS-PAGE separation and then transferred onto polyvinylidene fluoride membranes. The membranes were successively incubated with primary antibodies and horseradish peroxidase-conjugated secondary antibodies. Protein bands were visualized using the enhanced chemiluminescence detection reagent (Thermo Fisher Scientific).

For immunoprecipitation, cells were lysed on ice in a lysis buffer (50 mM Tris, 1% Triton X-100, 0.5 mM EDTA, 0.5 mM EGTA, 150 mM NaCl, 10% glycerol, pH 7.4) containing the protease inhibitor cocktail. The cell lysate was incubated with the appropriate antibodies overnight and then with protein A Sepharose beads (GE Healthcare Life Sciences). The immunoprecipitates were then subjected to immunoblotting.

### Quantitative RT-PCR analysis

Total RNAs were isolated from cells using the TRIzol reagent (Invitrogen) according to the manufacturer’s protocol. The RNAs were reverse transcribed using the Superscript III First Strand Synthesis System with oligo-dT primers (Invitrogen) and subjected to quantitative real-time PCR analysis in an Applied Bio systems 7500 HT Sequence Detection System using the Universal SYBR qPCR Master Mix (Vazyme). GAPDH or β-actin served as reference, and the 2^−ΔΔCT^ method was used to analyze the data. The primers used for quantitative RT-PCR are listed in S1 Table.

### Tyrosinase activity analysis

Cells were harvested by scraping, lysed on ice in a lysis buffer (phosphate buffer, 1% Triton X-100, pH 6.8) containing the protease inhibitor cocktail and centrifuged at 12,000 rpm for 10 min. The supernatant (90 μl) was mixed with 10 μl of 10 mM levodopa (Sigma-Aldrich) and was incubated at 37°C. The tyrosinase activity was measured by reading the absorbance at 475 nm using the Synergy H1 spectrophotometer (BioTek Instruments).

### Pull-down analysis

GST or GST-tagged proteins (cloned using the pGEX-6P-1 plasmid) were expressed in *E*. *coli* (BL21 strain) and resuspended in the GST buffer (150 mM NaCl, 1 mM MgCl2, 50 mM Hepes, 1 mM EGTA, and 0.5% Triton X-100, pH 7.4) containing the protease inhibitor PMSF. Ultrasonicated lysates were centrifuged at 12,000 rpm for 10 min and the supernatant was collected to be coupled with Glutathione Sepharose 4B beads (Amersham Bioscience) according to the manufacturer’s instructions. The beads were washed 3 times with the GST buffer and incubated with HEK293T cell lysates expressing MC1R-3×Flag or BBS2-HA for 4 h at 4°C. Then, the beads were pelleted and washed 5 times. The samples were then boiled and collected for immunoblotting and Coomassie brilliant blue staining.

### cAMP detection

For total intracellular cAMP level detection, A375 MC1R-KO cells rescued with MC1R or cilio-MC1R were plated in 12-well plate for 24 h, and then were pre-serum-starved and cultured in the absence of UV for 24 h before α-MSH treatment for the indicated time lapse. Then, cells were subjected to cAMP level detection with the human cAMP ELISA kit (LV10549, Animalunion Biotechnology) following the manufacturer’s protocol, and the optical density at 450 nm was determined.

In live cell ciliary cAMP assay, A375 MC1R-KO cells rescued with MC1R or cilio-MC1R were plated in glass bottom 96-well plate and transfected with the ratiometric cilium-targeted cADDis BacMam (Molecular Montana, D0211G) according to the manufacturer’s protocol. Cells were pre-serum-starved and cultured in the absence of UV for 24 h before α-MSH stimulation and live cell imaging under an SP8 confocal microscope (Leica) equipped with a 63×/1.40 numerical aperture objective lens. The single ciliated cell was videoed at 20 s intervals for 8 min. The cADDis is a downward cAMP reporter, whose green fluorescence decreased upon cAMP binding [33]. For ease of understanding, the cADDis signal was inverted to represent a direct relationship between cAMP level and cADDis intensity (1/cADDis). The 5HT6-mCherry signal was a constant mCherry fluorescence and was used to correct fluorescence artifact. The mCherry/cADDis ratio was therefore used to depict the dynamic changes in cAMP level, and the fluorescence intensities were calculated as: the fluorescence intensity of mCherry (red)/the fluorescence intensity of cADDis (green). The fluorescence intensity was measured with ImageJ.

cAMPinG1 is targeted to the cilium and plasma membrane by fusing with SSTR3. cAMPinG1 is a ratiometric cAMP reporter, whose fluorescence intensity upon binding to cAMP increases at 488 nm of excitation (ex488) and slightly decreases at 405 nm of excitation (ex405) [34], and 405 nm (emission detected between 475 and 525 nm) and 488 nm (emission detected between 510 and 560 nm) laser lines were used. Region of interest (ROI) for the cilium and plasma membrane was manually drawn and selected with ImageJ, and the fluorescence intensity of the ROI was measured.

### MST assay

HEK293T cells were transfected with WT or C315S MC1R-mEmerald and were then lysed on ice in a lysis buffer (50 mM Tris-HCl, 150 mM NaCl, 1% Triton X-100, pH 7.5) containing the protease inhibitor cocktail. Cell lysates were incubated with a series of dilutions (12 points) of PI4P (Echelon Biosciences) in the MST binding buffer (50 mM Tris-HCl, 150 mM NaCl, pH 7.5) for 30 min before being subjected to thermophoresis detection in a Monolith NT.115 instrument (NanoTemper Technologies), and data were analyzed using the NTAnalysis software (NanoTemper Technologies).

### Palmitoylation assay

Cells were lysed on ice in a lysis buffer (1% IGEPAL CA-630, 50 mM Tris-HCl, 150 mM NaCl 10% glycerol, 50 mM N-ethylmaleimide, pH 7.5) containing the protease inhibitor cocktail, and MC1R-mEmerald was immunoprecipitated with the GFP antibody. Purified MC1R-mEmerald was incubated with 1 M hydroxylamine (Sigma-Aldrich) for 1 h at room temperature and washed with the lysis buffer (pH 6.2) containing 2 μm biotin-BMCC (Thermo Fisher Scientific) for 1 h at 4°C. The samples were subjected to immunoblotting, and the level of protein palmitoylation was analyzed with horseradish peroxidase-conjugated streptavidin.

### Dual-luciferase reporter assay

The DNA fragment containing the *DCT* promoter region was cloned into the pGL3-basic reporter vector expressing the firefly luciferase. HEK293T cells were transfected with the reporter vectors and an internal control vector expressing renilla luciferase. At 24 h after transfection, the luciferase activity was analyzed with the Firefly & Renilla Luciferase Reporter Assay Kit (Dalian Meilun Biotechnology) according to the manufacturer’s instructions.

### Statistical analysis

Analysis of statistical significance was performed with the one-way ANOVA or unpaired two-tailed Student’s *t* tests, as indicated in the figure legends. *P*-values less than 0.05 were considered statistically significant.

## Supporting information

S1 Fig UV and α-MSH stimulate ciliogenesis in melanocytes and melanoma cells. (A) Heatmap of transcriptomes in UVB-exposed and control skins from the Gene Expression Omnibus (GEO GSE56754). Ciliogenesis-related genes listed in the Syscilia Gold Standard (SCGS) [66] with *p*-value <0.05 were selected. (B–D) Immunofluorescence images (B) and quantification of the percentage of ciliated cells (*n* = 3 independent experiments) (C) and cilium length (*n* ≥ 50 cells) (D) of primary human melanocytes that were mock-treated, or treated with UV, 100 nM α-MSH, or both. Cells were treated with α-MSH for 24 h in the absence of serum after UV exposure. Cells were stained with the Arl13b antibody (green) and DAPI (red, pseudocolor). Ciliated cells were marked with white arrows. Scale bar, 10 μm. (E–G) Immunofluorescence images (E) and quantification of the percentage of ciliated cells (*n* = 3 independent experiments) (F) and cilium length (*n* ≥ 50 cells) (G) of primary human melanocytes that were mock-treated, or treated with UV, 100 nM α-MSH, or both. Cells were treated with α-MSH for 24 h in the presence of serum after UV exposure. Cells were stained with the ace-Tubulin (red) and γ-Tubulin (green) antibodies. Nuclei were stained with DAPI (blue). Ciliated cells were marked with white arrows. Scale bar, 10 μm. (H) Immunofluorescence images of SK-MEL-2 cells that were mock-treated, or treated with UV, 100 nM α-MSH, or both. Cells were treated with α-MSH for 24 h in the absence of serum after UV exposure, under either vehicle (DMSO) or cytochalasin D (Cyto D) treatment conditions. Cells were stained with the Arl13b antibody (green) and DAPI (red, pseudocolor). Ciliated cells were marked with white arrows. Scale bar, 10 μm. (I) Immunofluorescence images of A375 cells that were mock-treated, or treated with UV, 100 nM α-MSH, or both. Cells were treated with α-MSH for 24 h in the absence of serum after UV exposure, under either vehicle (DMSO) or Cyto D treatment conditions. Cells were stained with the Arl13b antibody (green) and DAPI (red, pseudocolor). Ciliated cells were marked with white arrows. Scale bar, 10 μm. (J–L) Immunofluorescence images (J) and quantification of the percentage of ciliated cells (*n* = 3 independent experiments) (K) and cilium length (*n* ≥ 50 cells) (L) of A375 cells that were treated with vehicle (PBS) or 10 nM ASIP with or without UV/α-MSH (100 nM). Cells were treated with α-MSH and ASIP for 24 h in the absence of serum after UV exposure. Cells were stained with the Arl13b antibody (green) and DAPI (red, pseudocolor). Ciliated cells were marked with white arrows. Scale bar, 10 μm. Data are presented as mean ± SD. Statistical significance was determined by one-way ANOVA (C, D, F, and G) or unpaired two-tailed Student’s *t* test (K and L); *p < 0.05, **p < 0.01, ***p < 0.001; ns, not significant. The underlying data for this figure can be found in S3 File, S4 File, and S1 Data.
(TIF)

S2 Fig MC1R transcriptionally regulates ciliogenesis-related genes. (A) Immunoblotting showing the expression of MC1R in A375 MC1R-KO cells rescued with MC1R variants. β-actin served as a control. (B) Heatmap of differentially expressed ciliogenesis-related genes in A375 MC1R-KO cells rescued with the control vector (A375 MC1R-KO) or WT MC1R (A375 MC1R-WT). Cells were cultured in the absence of serum and treated with UV/α-MSH (100 nM). Cells were treated with α-MSH for 12 h after UV exposure and subjected to mRNA extraction. Only genes with absolute Log_2_|fold change| > 0.5 and adjusted *p*-value < 0.05 were considered differentially expressed. (C) Quantitative RT-PCR analysis of ciliogenesis-related genes selected from (B) in A375 MC1R-KO and A375 MC1R-WT cells treated with UV/α-MSH (100 nM). Cells were treated with α-MSH for 12 h in the absence of serum after UV exposure (*n* = 3 independent experiments). (D) Immunoblotting of ciliogenesis-related genes selected from (B) in A375 MC1R-KO and A375 MC1R-WT cells treated with UV/α-MSH (100 nM). Cells were treated with α-MSH for 36 h in the absence of serum after UV exposure. The band of TTBK2 was marked with red arrow. GAPDH served as a control. (E) Quantitative RT-PCR analysis of ciliogenesis-related genes selected from (B) in A375 MC1R-WT cells treated with UV alone. Cells were cultured in the absence of serum for 12 h after UV exposure (*n* = 3 independent experiments). Data are presented as mean ± SD. Statistical significance was determined with unpaired two-tailed Student’s *t* tests; **p < 0.01, ***p < 0.001; ns, not significant. The underlying data for this figure can be found in S1 Data. The uncropped blots are included in S1 Raw Images.
(TIF)

S3 Fig Ciliary localization of MC1R regulates its function in melanogenesis. (A, B) Immunofluorescence images (A) and quantification of the percentage of ciliated cells (*n* = 3 independent experiments) (B) of primary human melanocytes that were mock-treated, or treated with UV, 100 nM α-MSH, or both. Cells were treated with α-MSH for 24 h in the presence of serum after UV exposure. Cells were stained with the Arl13b antibody (green) and DAPI (red, pseudocolor). Scale bar, 10 μm. (C, D) Immunofluorescence images (C) and quantification of the percentage of ciliated cells (*n* = 3 independent experiments) (D) of A375 MC1R-KO cells rescued with WT MC1R (A375 MC1R-WT) that were mock-treated, or treated with UV, 100 nM α-MSH, or both. Cells were treated with α-MSH for 24 h in the absence of serum after UV exposure. Cells were stained with the Arl13b antibody (green) and DAPI (red, pseudocolor). Scale bar, 10 μm. (E) Tyrosinase activity of A375 MC1R-WT cells that were mock treated, treated with 100 nM α-MSH, 100 nM α-MSH/30 μm ciliobrevin A (Cilio A), or 100 nM α-MSH/2 mM chloral hydrate (CH) for 36 h in the absence of serum after UV exposure (*n* = 3 independent experiments). (F) Quantitative RT-PCR analysis of melanogenesis-related genes of A375 MC1R-KO or MC1R-WT cells treated as described in E (*n* = 3 independent experiments). (G) Immunoblot analysis of melanogenesis-related proteins in A375 MC1R-WT cells treated as described in E. GAPDH served as a control. (H) Immunofluorescence images of A375 MC1R-KO cells transfected with Arl13b-mEmerald, MC1R-mEmerald, WT MC1R-Arl13b-mEmerald, R151C MC1R-Arl13b-mEmerald, or R160W MC1R-Arl13b-mEmerald. Cells were serum starved for 36 h before fixing. Cells were stained with the ace-Tubulin antibody (red). Scale bar, 5 μm. (I) Quantification of the percentage of ciliated cells with ciliary localization of MC1R as shown in panel H (*n* = 3 independent experiments). Data are presented as mean ± SD. Statistical significance was determined with one-way ANOVA; *p < 0.05, ***p < 0.001. The underlying data for this figure can be found in S1 Data. The uncropped blots are included in S1 Raw Images.
(TIF)

S4 Fig Regulation of ciliary MC1R by BBSome proteins. (A, B) Immunofluorescence images (A) of A375 MC1R-KO cells rescued with MC1R-mEmerald (A375 MC1R-mEmerald, green) and transfected with the control vector or MRAP1-HA. Cells were treated with UV/α-MSH (100 nM). Cells were treated with α-MSH for 36 h in the absence of serum after UV exposure and were stained with the Arl13b antibody (red). The percentage of ciliated cells with ciliary localization of MC1R was quantified in panel B (*n* = 3 independent experiments). MC1R-localized cilia were marked with yellow arrows. Scale bar, 25 μm. (C, D) Immunofluorescence images (C) of A375 MC1R-KO cells rescued with MC1R-mEmerald (green) and transfected with the control vector or MRAP2-HA. Cells were treated with UV/α-MSH (100 nM). Cells were treated with α-MSH for 36 h in the absence of serum after UV exposure and were stained with the Arl13b antibody (red). The percentage of ciliated cells with ciliary localization of MC1R was quantified in panel D (*n* = 3 independent experiments). MC1R-localized cilia were marked with yellow arrows. Scale bar, 25 μm. (E, F) Immunofluorescence images (E) of A375 MC1R-KO cells rescued with MC1R-mEmerald (green) and transfected with the control vector or Tulp3-HA. Cells were treated with UV/α-MSH (100 nM). Cells were treated with α-MSH for 36 h in the absence of serum after UV exposure and were stained with the Arl13b antibody (red). The percentage of ciliated cells with ciliary localization of MC1R was quantified in panel F (*n* = 3 independent experiments). MC1R-localized cilia were marked with yellow arrows. Scale bar, 25 μm. (G) Immunoblotting showing the expression of Tulp3 in cell lines. GAPDH served as a control. (H) Immunoprecipitation and immunoblotting showing the interaction between MC1R-3×Flag and HA-tagged BBSome proteins. HEK293T cells were co-transfected with the indicated plasmids, and immunoprecipitation was performed with the HA antibody. (I) Topological diagram of MC1R. EL, extracellular loop; IL, intracellular loop. (J) Immunoblotting showing the efficiency of BBS2 knockdown in A375 MC1R-mEmerald cells. Cells were transfected with control or BBS2 shRNAs in the presence or absence of BBS2-HA. (K, L) Immunofluorescence images (K) of A375 MC1R-KO cells rescued with MC1R-mEmerald (green). Cells were transfected with control, BBS1, or BBS5 shRNAs, the sequences of which were previously reported [67,68]. Cells were treated with α-MSH (100 nM) for 36 h in the absence of serum after UV exposure and were stained with the Arl13b antibody (red). The percentage of ciliated cells with ciliary localization of MC1R was quantified in panel L (*n* = 3 independent experiments). Scale bar, 5 μm. (M) Immunoblotting showing the efficiency of BBS1 and BBS5 knockdown in A375 MC1R-mEmerald cells. Cells were transfected with control, BBS1, or BBS5 shRNAs. Data are presented as mean ± SD. Statistical significance was determined by unpaired two-tailed Student’s *t* test (B, D, and F) or one-way ANOVA (L); **p < 0.01, ***p < 0.001; ns, not significant. The underlying data for this figure can be found in S1 Data. The uncropped blots are included in S1 Raw Images.
(TIF)

S5 Fig Palmitoylation of MC1R and its interaction with BBS2. (A) Acyl-biotin exchange analysis of the level of MC1R palmitoylation in A375 MC1R-KO cells rescued with MC1R-mEmerald (A375 MC1R-mEmerald). Cells were treated with vehicle (DMSO), 100 nM α-MSH, 100 nM α-MSH/25 μm 2-bromopalmitate (2-BP), 100 nM α-MSH/1 μm palmostatin B (PalB), or 100 nM α-MSH/100 μm palmitic acid (PA) for 36 h in the absence of serum after UV exposure. (B) Immunoprecipitation and immunoblotting showing the interaction of MC1R mutants with BBS2. HEK293T cells were transfected with WT or C315S MC1R-3×Flag and BBS2-HA. Cell lysates were immunoprecipitated with the Flag antibody. The uncropped blots are included in S1 Raw Images.
(TIF)

S6 Fig Cilium-specific functions of MC1R. (A) Detection of ciliary cAMP levels using cilium-targeted cAMPinG1, which is targeted to the cilium and plasma membrane by fusing with SSTR3. A375 MC1R-KO cells rescued with MC1R or cilio-MC1R (constructed by fusing the ciliary protein Arl13b with MC1R) were pre-serum-starved and cultured in the absence of UV for 24 h. The fluorescence intensity was captured before ligand stimulation, 2 min after adding 1 μm α-MSH, and then 1 min after adding 100 μm forskolin. The cilium was marked with white arrows, and the plasma membrane was marked with red arrows. Scale bar, 10 μm. (B, C) Quantification of cAMP levels in the cilium (B) and at the plasma membrane (C) from panel A. cAMP levels were calculated as: the fluorescence intensity at 488 nm excitation/the fluorescence intensity at 405 nm excitation (ex488/ex405); *n* = 5 ciliated cells from 5 independent experiments. (D) Immunofluorescence images of A375 MC1R-KO cells transfected with Arl13b-mEmerald (control vector), MC1R-Arl13b-mEmerald (cilio-MC1R), or MC1R-Arl13b V359A-mEmerald (ciliary localization-deficient MC1R) (green). Cells were serum starved for 24 h before fixing and stained with the ace-Tubulin antibody (red). Scale bar, 5 μm. (E) Quantitative RT-PCR analysis of ciliogenesis-related genes in A375 MC1R-KO cells transfected with control vector, cilio-MC1R, or ciliary localization-deficient MC1R. Cells were treated with UV/α-MSH (100 nM). Cells were treated with α-MSH for 24 h in the absence of serum after UV exposure (*n* = 3 independent experiments). Data are presented as mean ± SD. Statistical significance was determined by unpaired two-tailed Student’s *t* test (B and C) or one-way ANOVA (E); **p < 0.01; ***p < 0.001; ns, not significant. The underlying data for this figure can be found in S1 Data.
(TIF)

S1 File Data underlying Fig 1B, 1E, and 1H. (PDF)

S2 File Data underlying Fig 2A and 2D. (PDF)

S3 File Data underlying S1B, S1E, and S1H Fig. (PDF)

S4 File Data underlying S1J and S1I Figs. (PDF)

S1 Data Excel file with all individual numerical values corresponding to the data presented in the main and supporting figures. Corresponding figure numbers are indicated in each Excel worksheet.
(XLSX)

S1 Raw Images PDF file with all uncropped gels and blots corresponding to the data presented in the main and supporting figures. (PDF)

S1 Table The primers used for quantitative RT-PCR. (XLSX)

## Abbreviations

α-MSH: α-melanocyte-stimulating hormone
ASIP: agouti-signaling protein
BBS: Bardet–Biedl syndrome
CREB: cAMP-response element binding protein
GEO: Gene Expression Omnibus
GO: gene ontology
GPCR: G protein-coupled receptor
IFT: intraflagellar transport
MC1R: melanocortin 1 receptor
MITF: microphthalmia-associated transcription factor
MRAP2: melanocortin 2 receptor accessory protein 2
MST: microscale thermophoresis
RHC: red hair color
ROI: region of interest
PKA: protein kinase A
TYRP1: tyrosinase related protein 1
UV: ultraviolet
