# Supplementary figures and images for: Melanocortin 1 receptor mediates melanin production by interacting with the BBSome in primary cilia
Source: PLoS Biol. 2024 Dec 2;22(12):e3002940. doi: 10.1371/journal.pbio.3002940 (PMC11637432; doi:10.1371/journal.pbio.3002940)

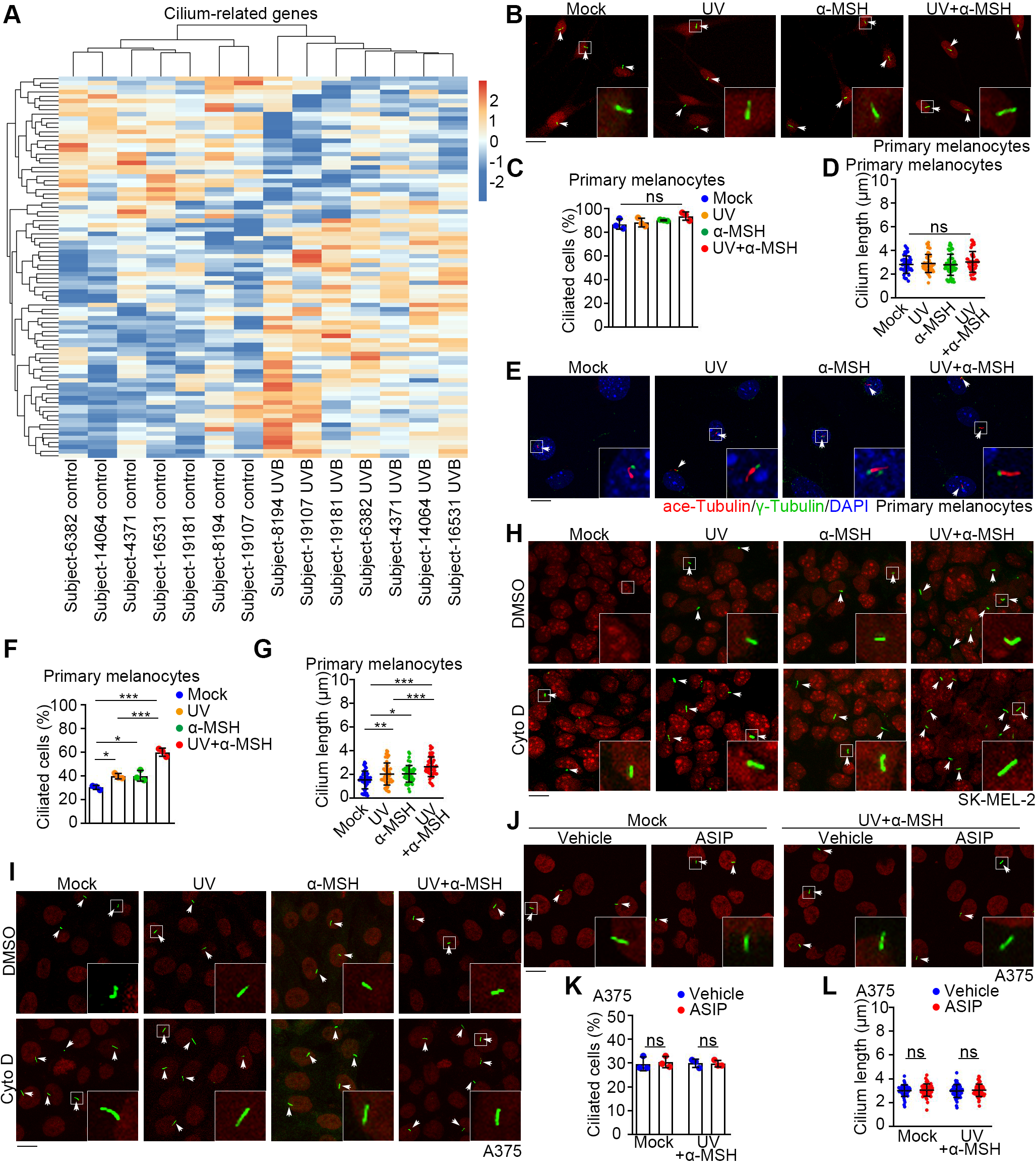

Supplement: S1 Fig — (A) Heatmap of transcriptomes in UVB-exposed and control skins from the Gene Expression Omnibus (GEO GSE56754). Ciliogenesis-related genes listed in the Syscilia Gold Standard (SCGS) [66] with p-value <0.05 were selected. (B–D) Immunofluorescence images (B) and quantification of the percentage of ciliated cells (n = 3 independent experiments) (C) and cilium length (n ≥ 50 cells) (D) of primary human melanocytes that were mock-treated, or treated with UV, 100 nM α-MSH, or both. Cells were treated with α-MSH for 24 h in the absence of serum after UV exposure. Cells were stained with the Arl13b antibody (green) and DAPI (red, pseudocolor). Ciliated cells were marked with white arrows. Scale bar, 10 μm. (E–G) Immunofluorescence images (E) and quantification of the percentage of ciliated cells (n = 3 independent experiments) (F) and cilium length (n ≥ 50 cells) (G) of primary human melanocytes that were mock-treated, or treated with UV, 100 nM α-MSH, or both. Cells were treated with α-MSH for 24 h in the presence of serum after UV exposure. Cells were stained with the ace-Tubulin (red) and γ-Tubulin (green) antibodies. Nuclei were stained with DAPI (blue). Ciliated cells were marked with white arrows. Scale bar, 10 μm. (H) Immunofluorescence images of SK-MEL-2 cells that were mock-treated, or treated with UV, 100 nM α-MSH, or both. Cells were treated with α-MSH for 24 h in the absence of serum after UV exposure, under either vehicle (DMSO) or cytochalasin D (Cyto D) treatment conditions. Cells were stained with the Arl13b antibody (green) and DAPI (red, pseudocolor). Ciliated cells were marked with white arrows. Scale bar, 10 μm. (I) Immunofluorescence images of A375 cells that were mock-treated, or treated with UV, 100 nM α-MSH, or both. Cells were treated with α-MSH for 24 h in the absence of serum after UV exposure, under either vehicle (DMSO) or Cyto D treatment conditions. Cells were stained with the Arl13b antibody (green) and DAPI (red, pseudocolor). Ciliated cell [file pbio.3002940.s001.tif]

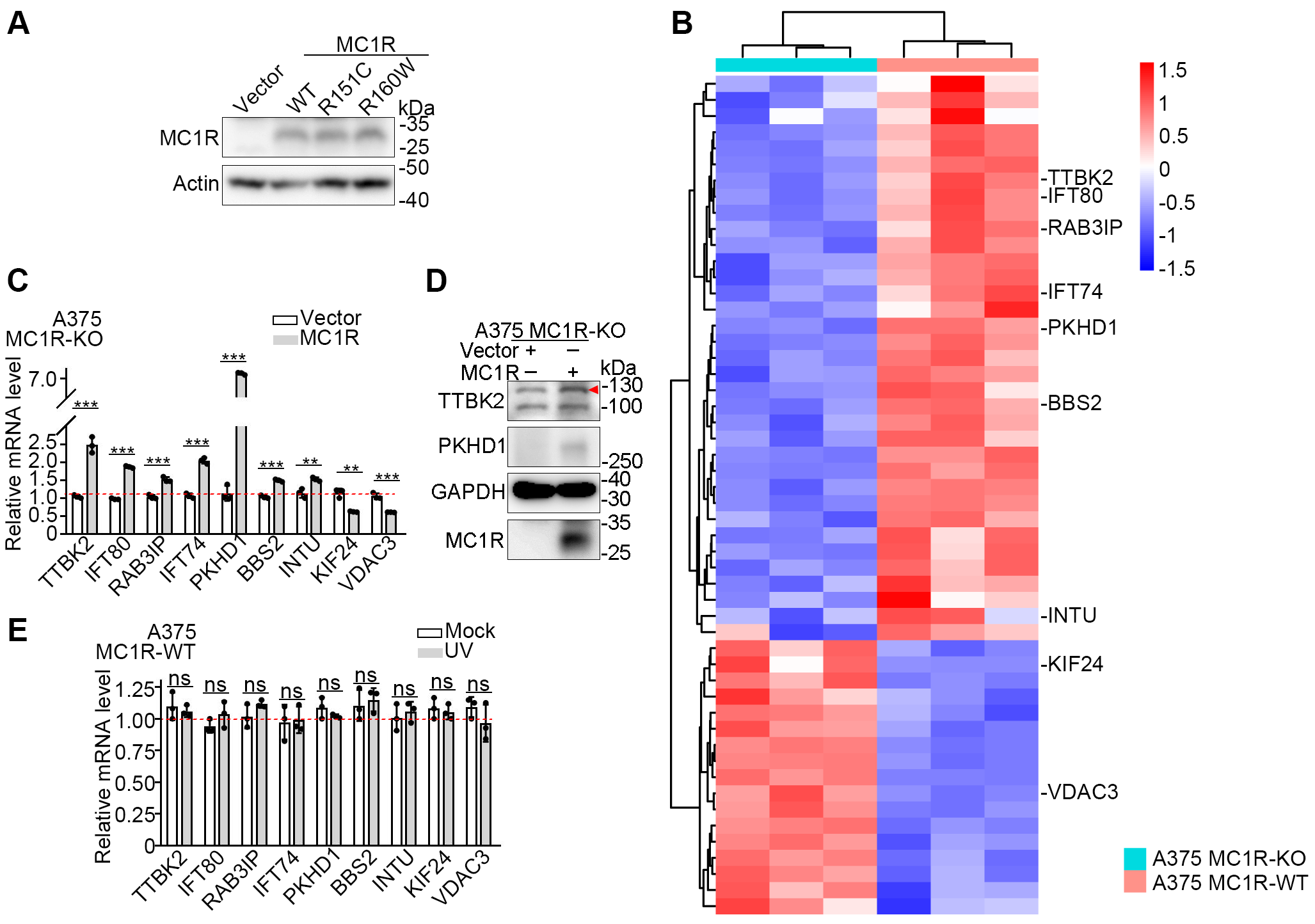

Supplement: S2 Fig — (A) Immunoblotting showing the expression of MC1R in A375 MC1R-KO cells rescued with MC1R variants. β-actin served as a control. (B) Heatmap of differentially expressed ciliogenesis-related genes in A375 MC1R-KO cells rescued with the control vector (A375 MC1R-KO) or WT MC1R (A375 MC1R-WT). Cells were cultured in the absence of serum and treated with UV/α-MSH (100 nM). Cells were treated with α-MSH for 12 h after UV exposure and subjected to mRNA extraction. Only genes with absolute Log2|fold change| > 0.5 and adjusted p-value < 0.05 were considered differentially expressed. (C) Quantitative RT-PCR analysis of ciliogenesis-related genes selected from (B) in A375 MC1R-KO and A375 MC1R-WT cells treated with UV/α-MSH (100 nM). Cells were treated with α-MSH for 12 h in the absence of serum after UV exposure (n = 3 independent experiments). (D) Immunoblotting of ciliogenesis-related genes selected from (B) in A375 MC1R-KO and A375 MC1R-WT cells treated with UV/α-MSH (100 nM). Cells were treated with α-MSH for 36 h in the absence of serum after UV exposure. The band of TTBK2 was marked with red arrow. GAPDH served as a control. (E) Quantitative RT-PCR analysis of ciliogenesis-related genes selected from (B) in A375 MC1R-WT cells treated with UV alone. Cells were cultured in the absence of serum for 12 h after UV exposure (n = 3 independent experiments). Data are presented as mean ± SD. Statistical significance was determined with unpaired two-tailed Student’s t tests; **p < 0.01, ***p < 0.001; ns, not significant. The underlying data for this figure can be found in S1 Data. The uncropped blots are included in S1 Raw Images. (TIF) [file pbio.3002940.s002.tif]

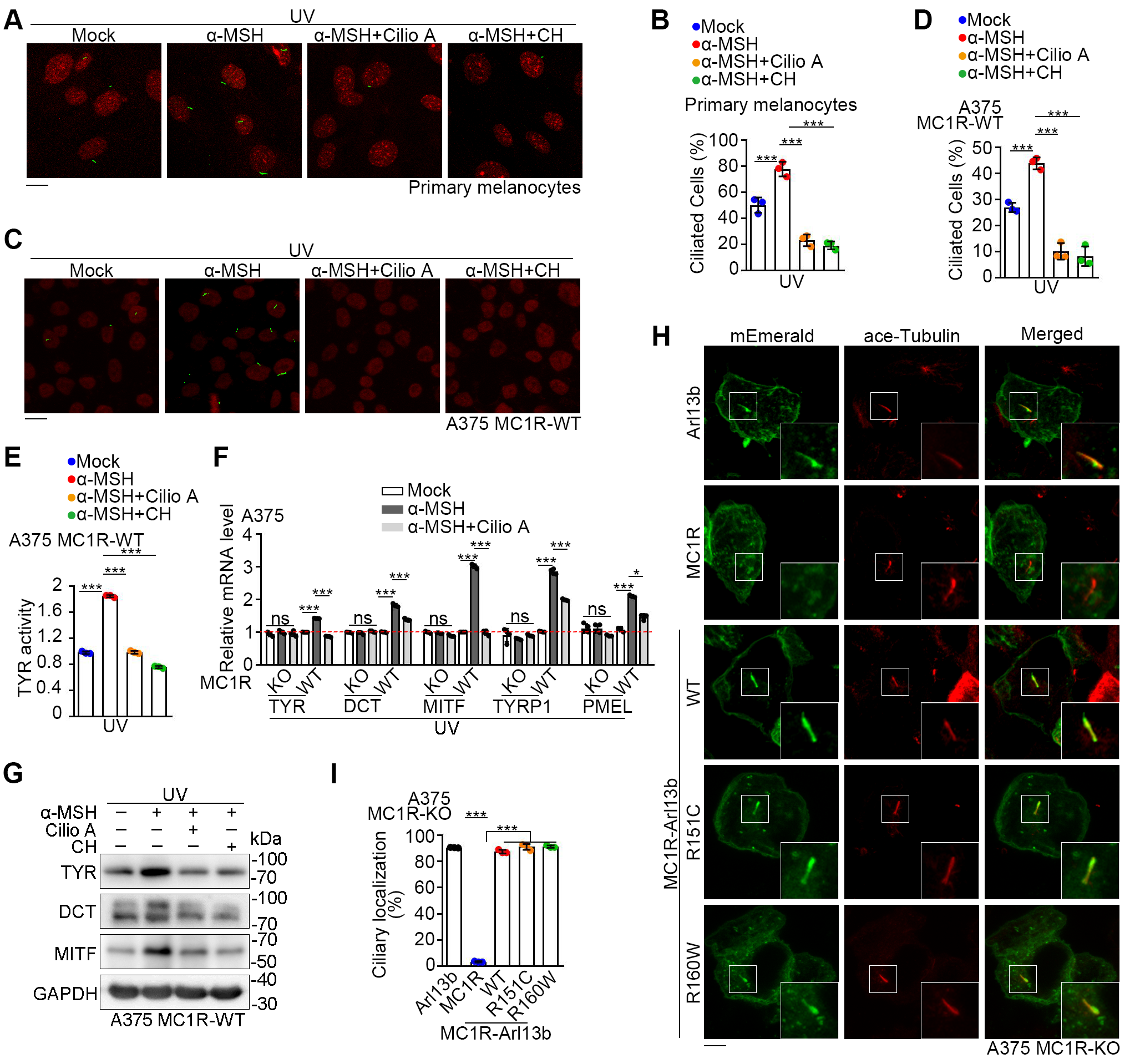

Supplement: S3 Fig — (A, B) Immunofluorescence images (A) and quantification of the percentage of ciliated cells (n = 3 independent experiments) (B) of primary human melanocytes that were mock-treated, or treated with UV, 100 nM α-MSH, or both. Cells were treated with α-MSH for 24 h in the presence of serum after UV exposure. Cells were stained with the Arl13b antibody (green) and DAPI (red, pseudocolor). Scale bar, 10 μm. (C, D) Immunofluorescence images (C) and quantification of the percentage of ciliated cells (n = 3 independent experiments) (D) of A375 MC1R-KO cells rescued with WT MC1R (A375 MC1R-WT) that were mock-treated, or treated with UV, 100 nM α-MSH, or both. Cells were treated with α-MSH for 24 h in the absence of serum after UV exposure. Cells were stained with the Arl13b antibody (green) and DAPI (red, pseudocolor). Scale bar, 10 μm. (E) Tyrosinase activity of A375 MC1R-WT cells that were mock treated, treated with 100 nM α-MSH, 100 nM α-MSH/30 μm ciliobrevin A (Cilio A), or 100 nM α-MSH/2 mM chloral hydrate (CH) for 36 h in the absence of serum after UV exposure (n = 3 independent experiments). (F) Quantitative RT-PCR analysis of melanogenesis-related genes of A375 MC1R-KO or MC1R-WT cells treated as described in E (n = 3 independent experiments). (G) Immunoblot analysis of melanogenesis-related proteins in A375 MC1R-WT cells treated as described in E. GAPDH served as a control. (H) Immunofluorescence images of A375 MC1R-KO cells transfected with Arl13b-mEmerald, MC1R-mEmerald, WT MC1R-Arl13b-mEmerald, R151C MC1R-Arl13b-mEmerald, or R160W MC1R-Arl13b-mEmerald. Cells were serum starved for 36 h before fixing. Cells were stained with the ace-Tubulin antibody (red). Scale bar, 5 μm. (I) Quantification of the percentage of ciliated cells with ciliary localization of MC1R as shown in panel H (n = 3 independent experiments). Data are presented as mean ± SD. Statistical significance was determined with one-way ANOVA; *p < 0.05, ***p < 0.001. The underlying data for this figure [file pbio.3002940.s003.tif]

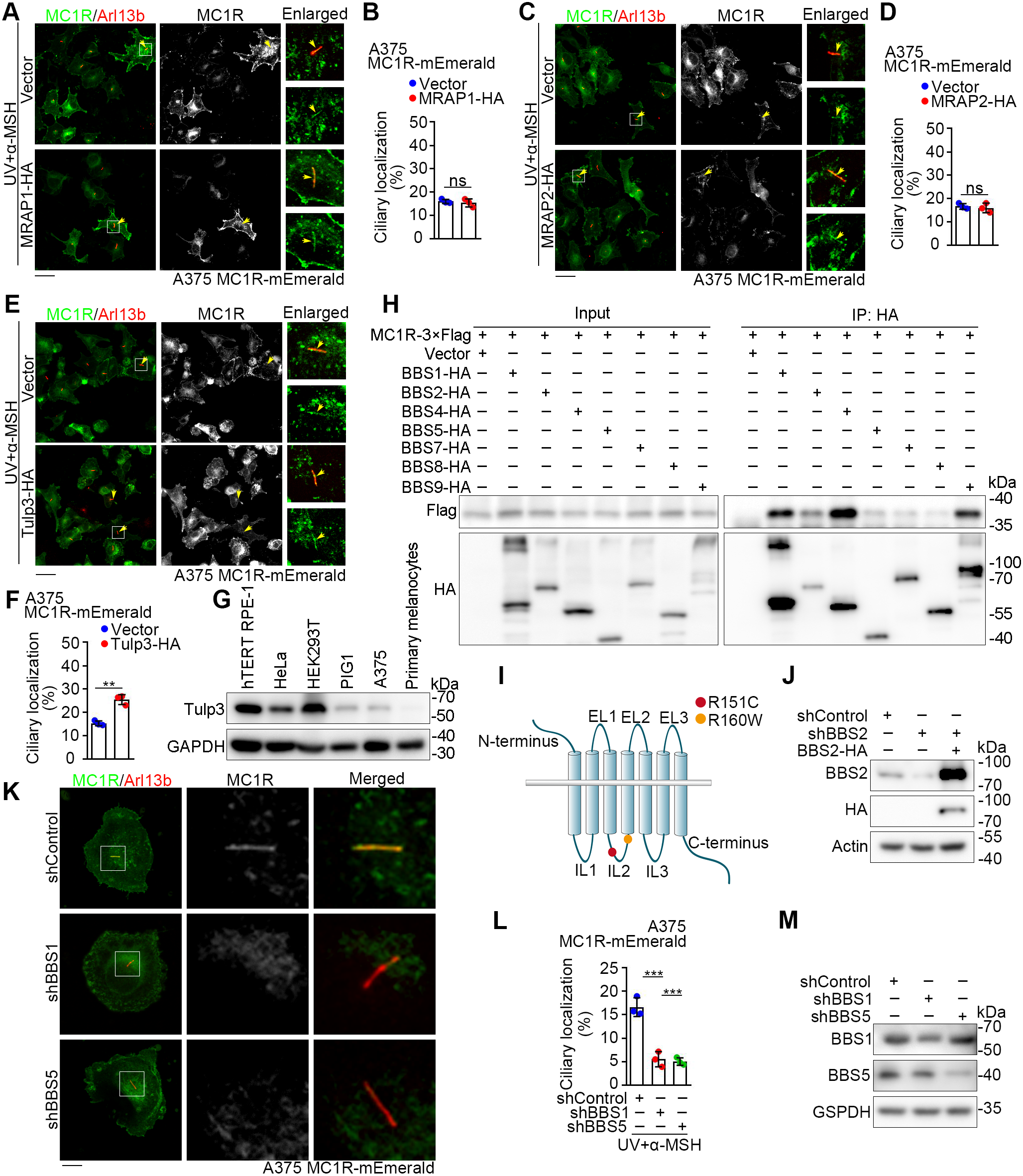

Supplement: S4 Fig — (A, B) Immunofluorescence images (A) of A375 MC1R-KO cells rescued with MC1R-mEmerald (A375 MC1R-mEmerald, green) and transfected with the control vector or MRAP1-HA. Cells were treated with UV/α-MSH (100 nM). Cells were treated with α-MSH for 36 h in the absence of serum after UV exposure and were stained with the Arl13b antibody (red). The percentage of ciliated cells with ciliary localization of MC1R was quantified in panel B (n = 3 independent experiments). MC1R-localized cilia were marked with yellow arrows. Scale bar, 25 μm. (C, D) Immunofluorescence images (C) of A375 MC1R-KO cells rescued with MC1R-mEmerald (green) and transfected with the control vector or MRAP2-HA. Cells were treated with UV/α-MSH (100 nM). Cells were treated with α-MSH for 36 h in the absence of serum after UV exposure and were stained with the Arl13b antibody (red). The percentage of ciliated cells with ciliary localization of MC1R was quantified in panel D (n = 3 independent experiments). MC1R-localized cilia were marked with yellow arrows. Scale bar, 25 μm. (E, F) Immunofluorescence images (E) of A375 MC1R-KO cells rescued with MC1R-mEmerald (green) and transfected with the control vector or Tulp3-HA. Cells were treated with UV/α-MSH (100 nM). Cells were treated with α-MSH for 36 h in the absence of serum after UV exposure and were stained with the Arl13b antibody (red). The percentage of ciliated cells with ciliary localization of MC1R was quantified in panel F (n = 3 independent experiments). MC1R-localized cilia were marked with yellow arrows. Scale bar, 25 μm. (G) Immunoblotting showing the expression of Tulp3 in cell lines. GAPDH served as a control. (H) Immunoprecipitation and immunoblotting showing the interaction between MC1R-3×Flag and HA-tagged BBSome proteins. HEK293T cells were co-transfected with the indicated plasmids, and immunoprecipitation was performed with the HA antibody. (I) Topological diagram of MC1R. EL, extracellular loop; IL, intracellular loop. (J) Immunoblot [file pbio.3002940.s004.tif]

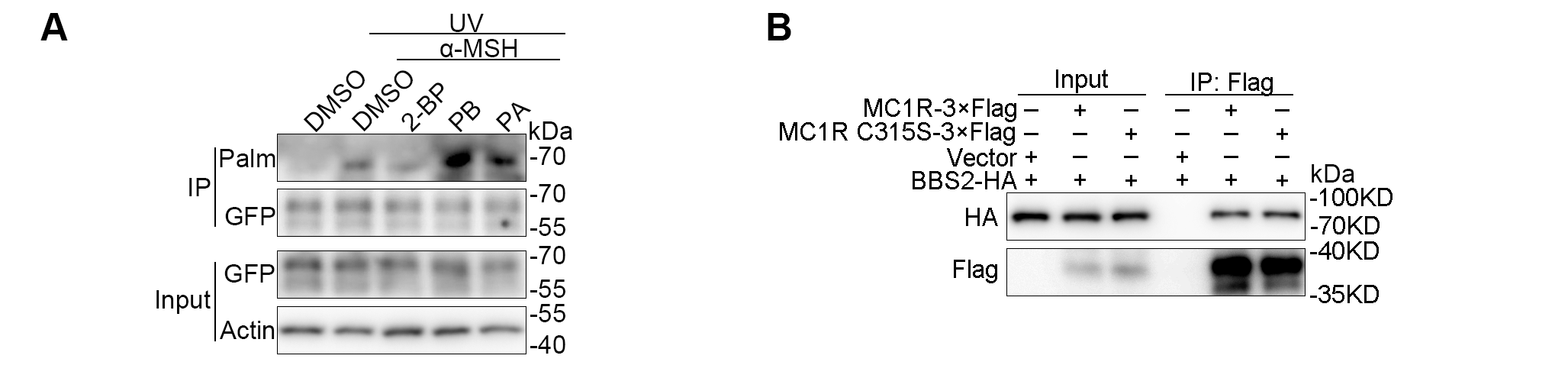

Supplement: S5 Fig — (A) Acyl-biotin exchange analysis of the level of MC1R palmitoylation in A375 MC1R-KO cells rescued with MC1R-mEmerald (A375 MC1R-mEmerald). Cells were treated with vehicle (DMSO), 100 nM α-MSH, 100 nM α-MSH/25 μm 2-bromopalmitate (2-BP), 100 nM α-MSH/1 μm palmostatin B (PalB), or 100 nM α-MSH/100 μm palmitic acid (PA) for 36 h in the absence of serum after UV exposure. (B) Immunoprecipitation and immunoblotting showing the interaction of MC1R mutants with BBS2. HEK293T cells were transfected with WT or C315S MC1R-3×Flag and BBS2-HA. Cell lysates were immunoprecipitated with the Flag antibody. The uncropped blots are included in S1 Raw Images. (TIF) [file pbio.3002940.s005.tif]

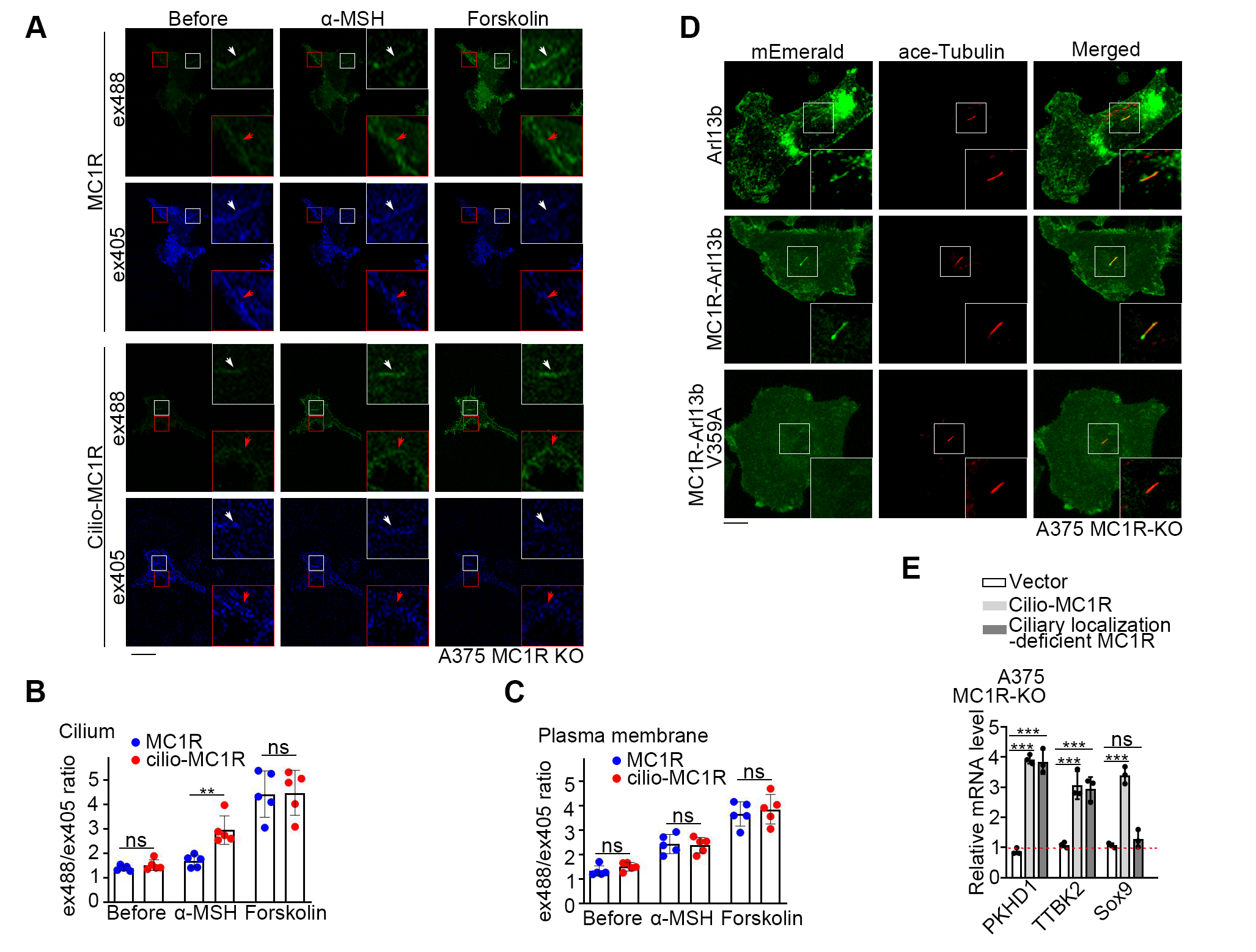

Supplement: S6 Fig — (A) Detection of ciliary cAMP levels using cilium-targeted cAMPinG1, which is targeted to the cilium and plasma membrane by fusing with SSTR3. A375 MC1R-KO cells rescued with MC1R or cilio-MC1R (constructed by fusing the ciliary protein Arl13b with MC1R) were pre-serum-starved and cultured in the absence of UV for 24 h. The fluorescence intensity was captured before ligand stimulation, 2 min after adding 1 μm α-MSH, and then 1 min after adding 100 μm forskolin. The cilium was marked with white arrows, and the plasma membrane was marked with red arrows. Scale bar, 10 μm. (B, C) Quantification of cAMP levels in the cilium (B) and at the plasma membrane (C) from panel A. cAMP levels were calculated as: the fluorescence intensity at 488 nm excitation/the fluorescence intensity at 405 nm excitation (ex488/ex405); n = 5 ciliated cells from 5 independent experiments. (D) Immunofluorescence images of A375 MC1R-KO cells transfected with Arl13b-mEmerald (control vector), MC1R-Arl13b-mEmerald (cilio-MC1R), or MC1R-Arl13b V359A-mEmerald (ciliary localization-deficient MC1R) (green). Cells were serum starved for 24 h before fixing and stained with the ace-Tubulin antibody (red). Scale bar, 5 μm. (E) Quantitative RT-PCR analysis of ciliogenesis-related genes in A375 MC1R-KO cells transfected with control vector, cilio-MC1R, or ciliary localization-deficient MC1R. Cells were treated with UV/α-MSH (100 nM). Cells were treated with α-MSH for 24 h in the absence of serum after UV exposure (n = 3 independent experiments). Data are presented as mean ± SD. Statistical significance was determined by unpaired two-tailed Student’s t test (B and C) or one-way ANOVA (E); **p < 0.01; ***p < 0.001; ns, not significant. The underlying data for this figure can be found in S1 Data. (TIF) [file pbio.3002940.s006.tif]

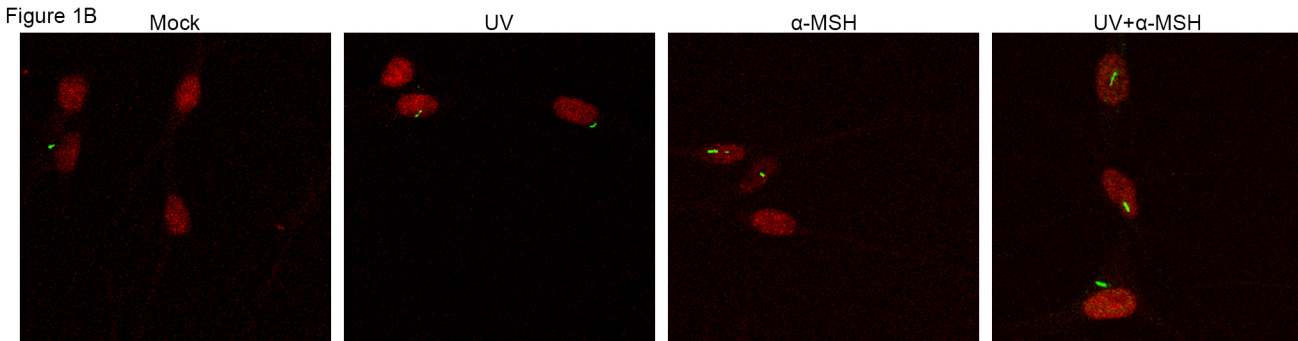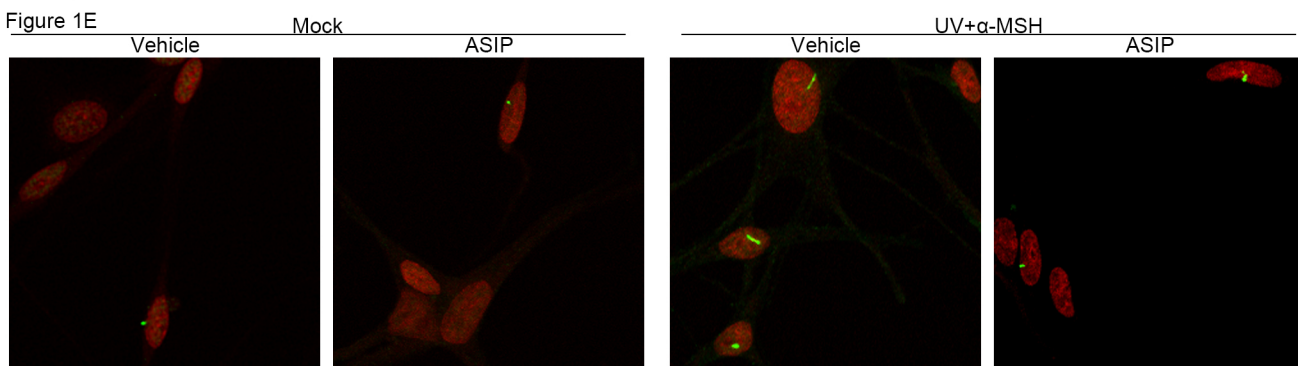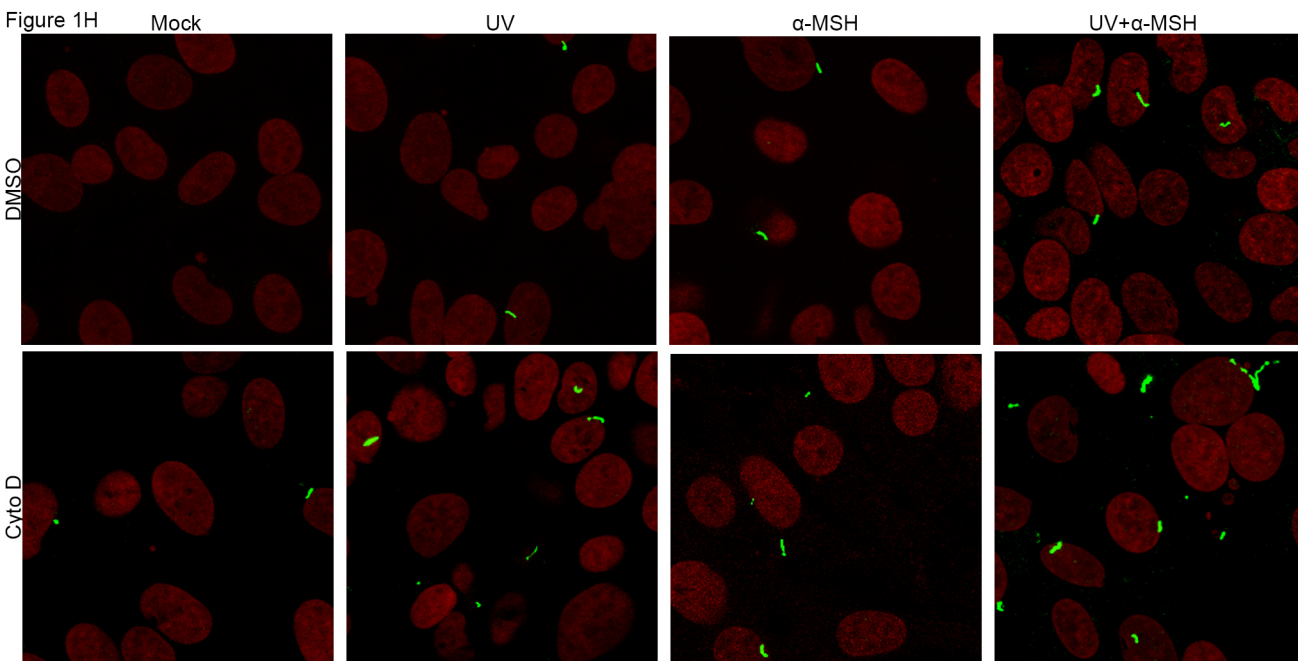

Supplement: S1 File — (PDF) [file pbio.3002940.s007.pdf]

Figure 2A

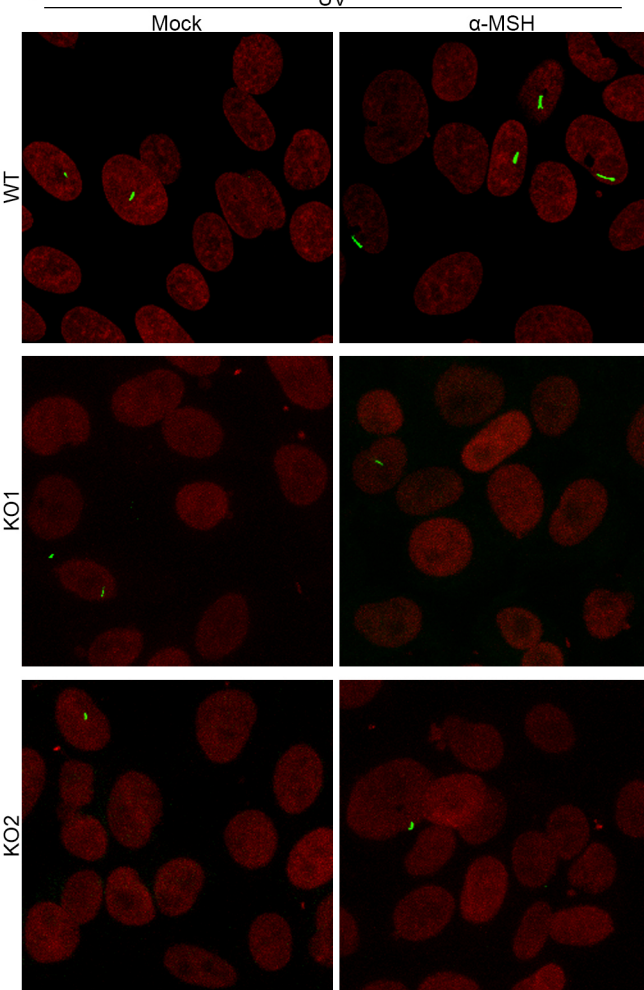

Figure 2D

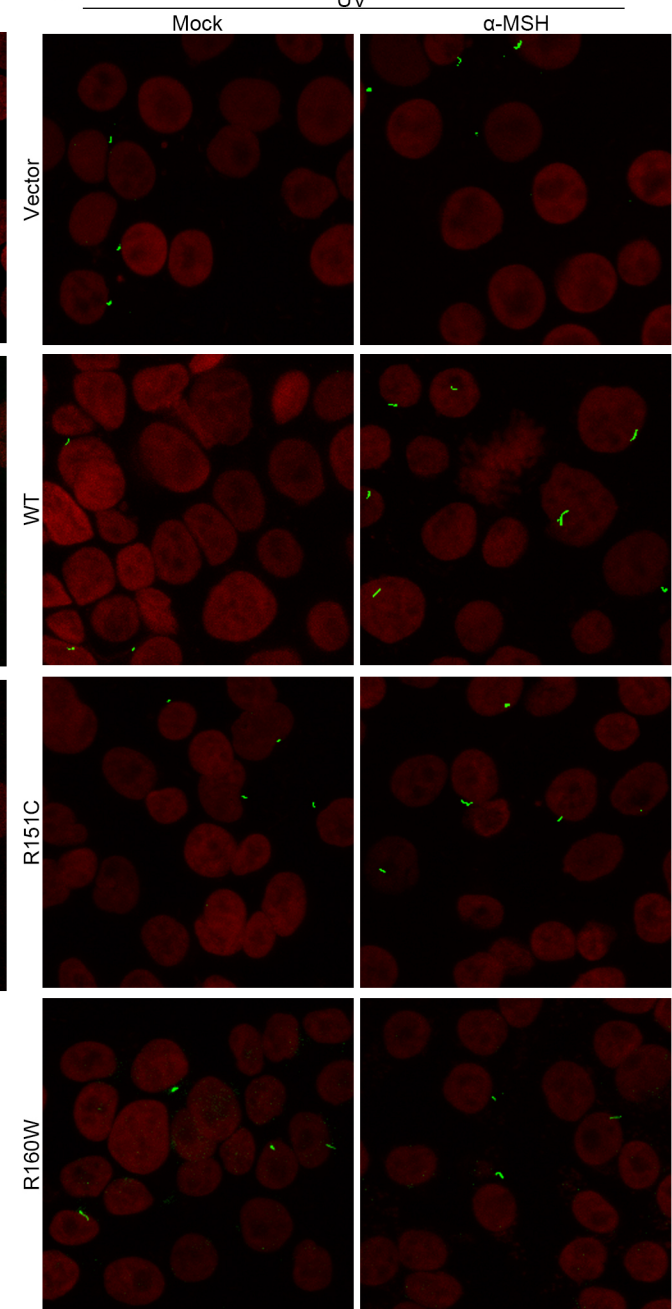

Supplement: S2 File — (PDF) [file pbio.3002940.s008.pdf]

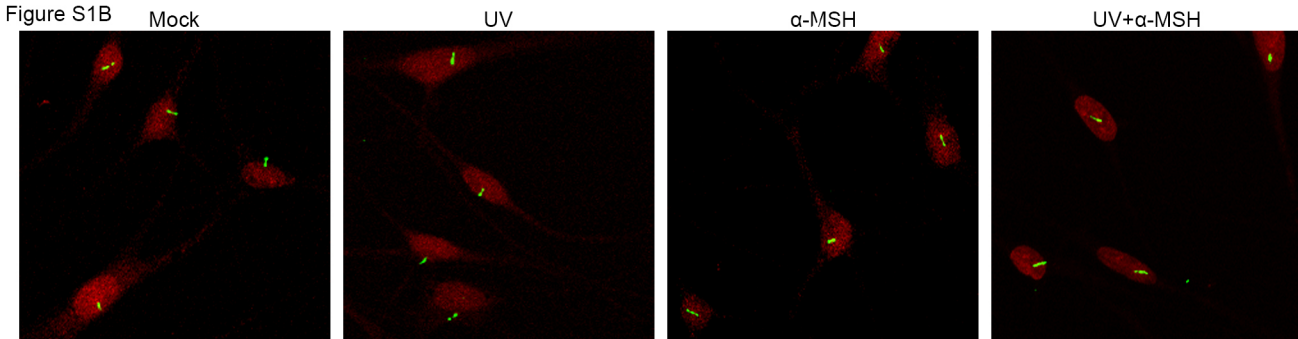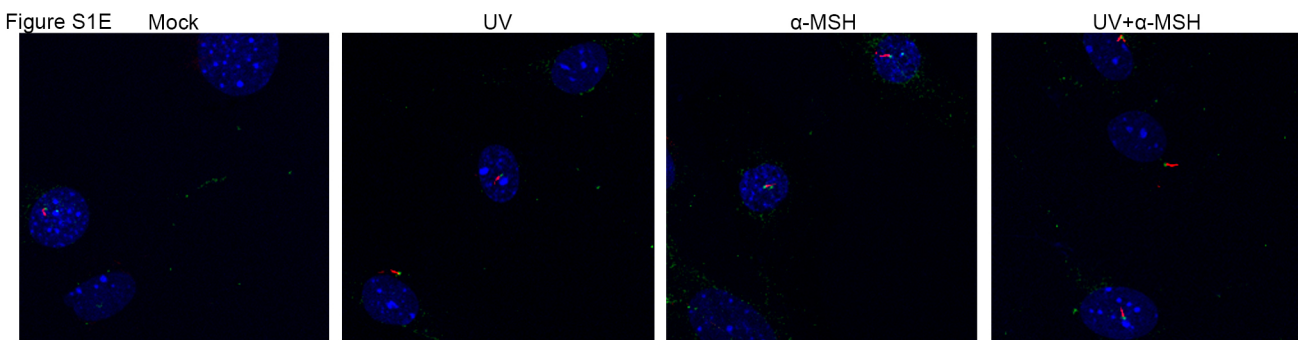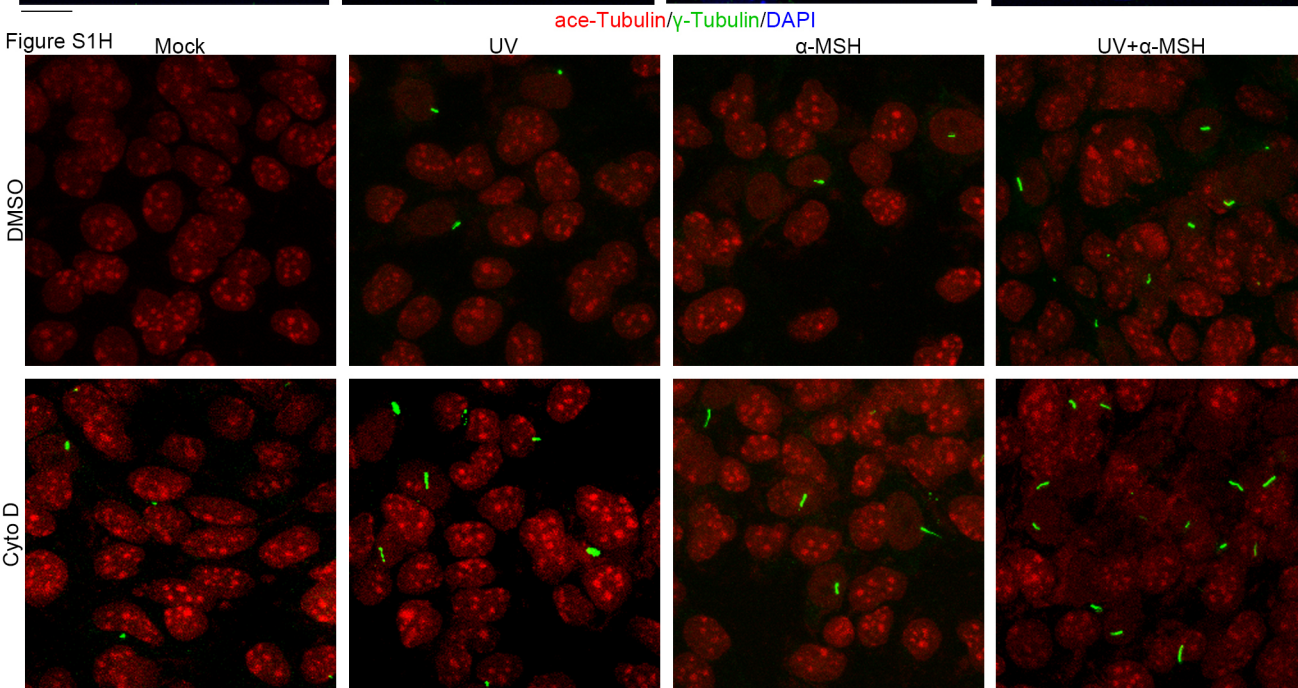

Supplement: S3 File — (PDF) [file pbio.3002940.s009.pdf]

Figure S1J

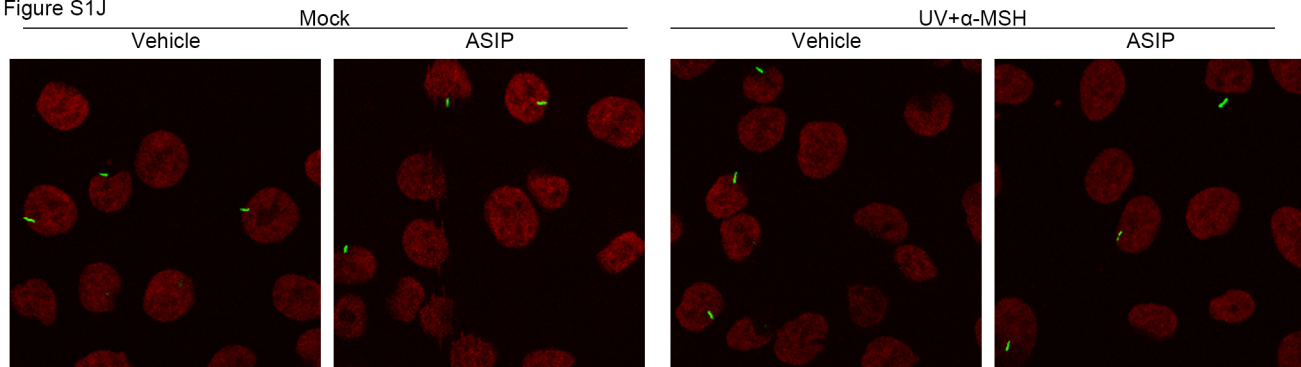

Figure S1I

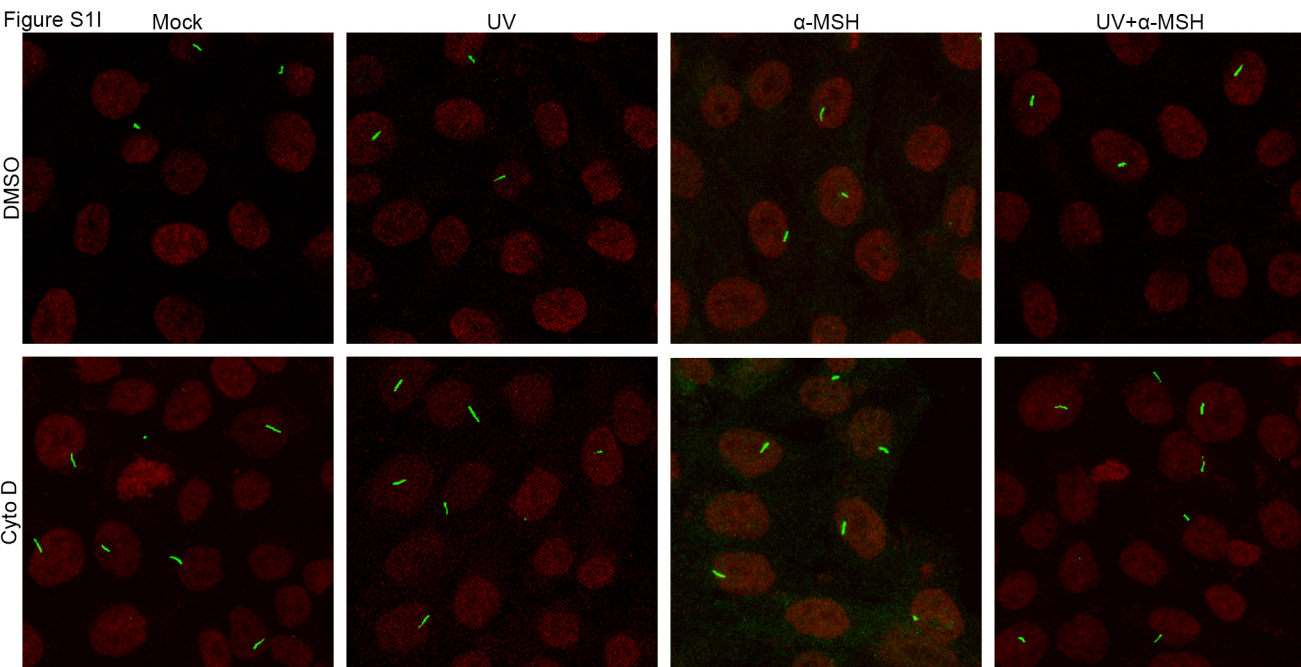

Supplement: S4 File — (PDF) [file pbio.3002940.s010.pdf]
