# Supplementary material for: Melanocortin 1 receptor mediates melanin production by interacting with the BBSome in primary cilia
Source: PLoS Biol. 2024 Dec 2;22(12):e3002940. doi: 10.1371/journal.pbio.3002940 (PMC11637432; doi:10.1371/journal.pbio.3002940)

Figure 3I

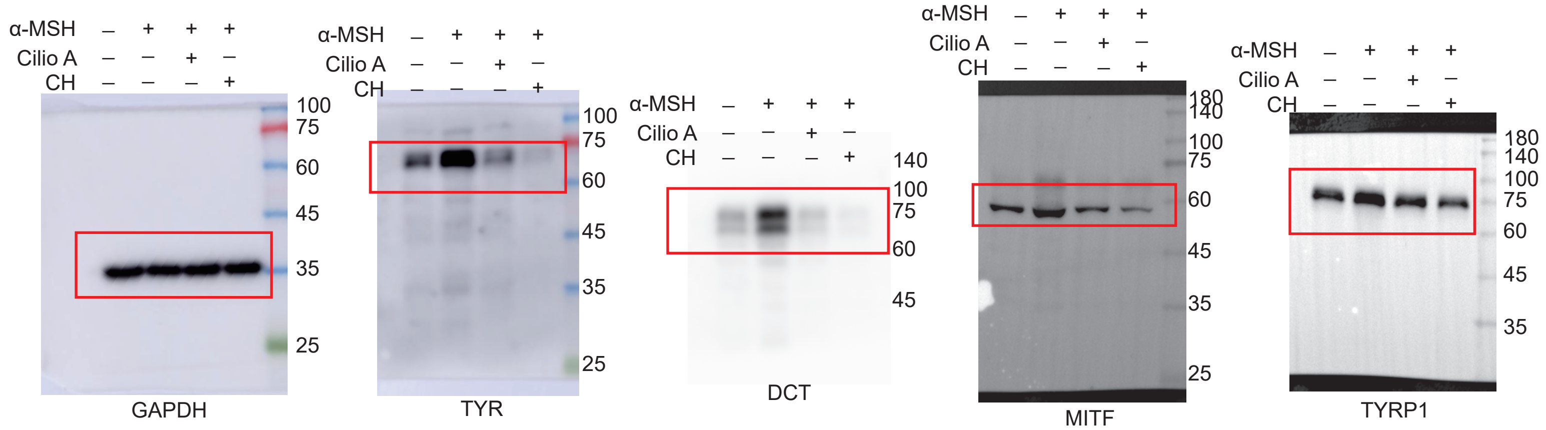

Figure 4A

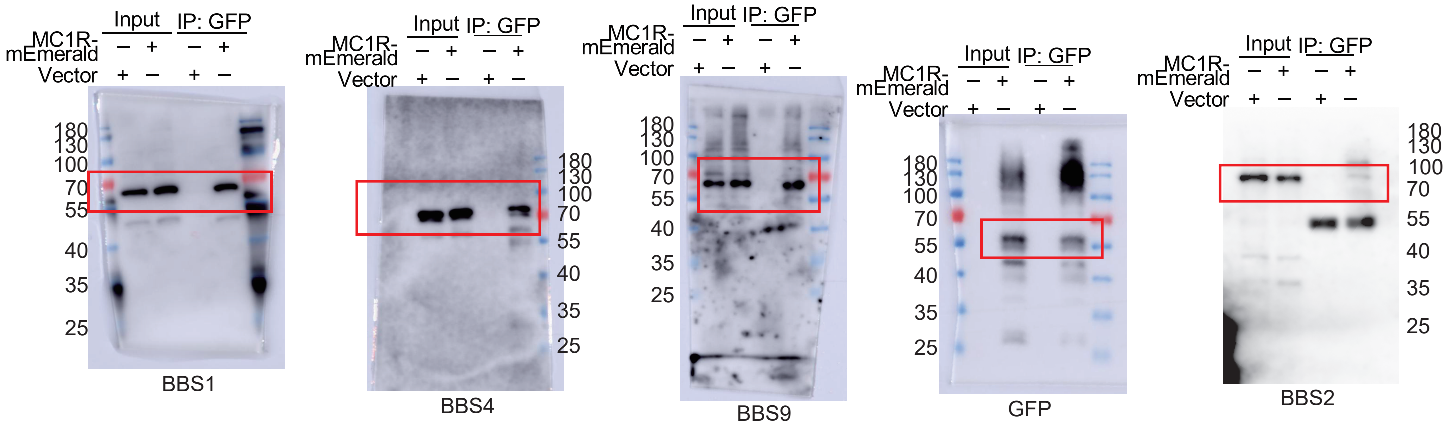

Figure 4B

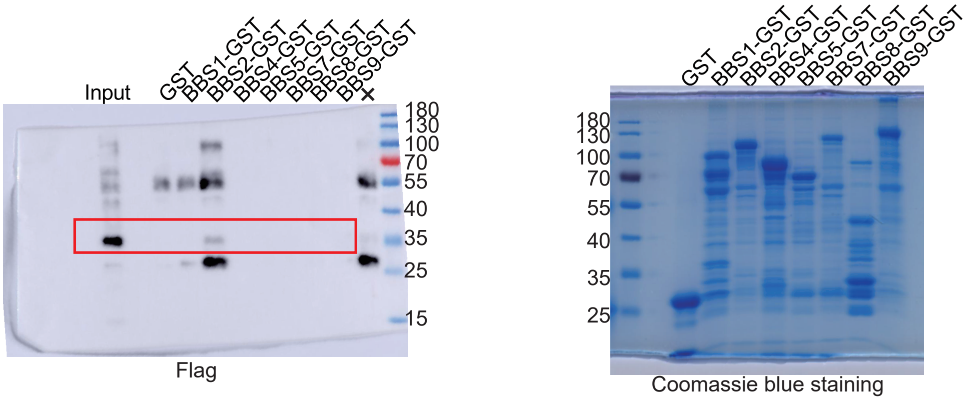

Figure 4C

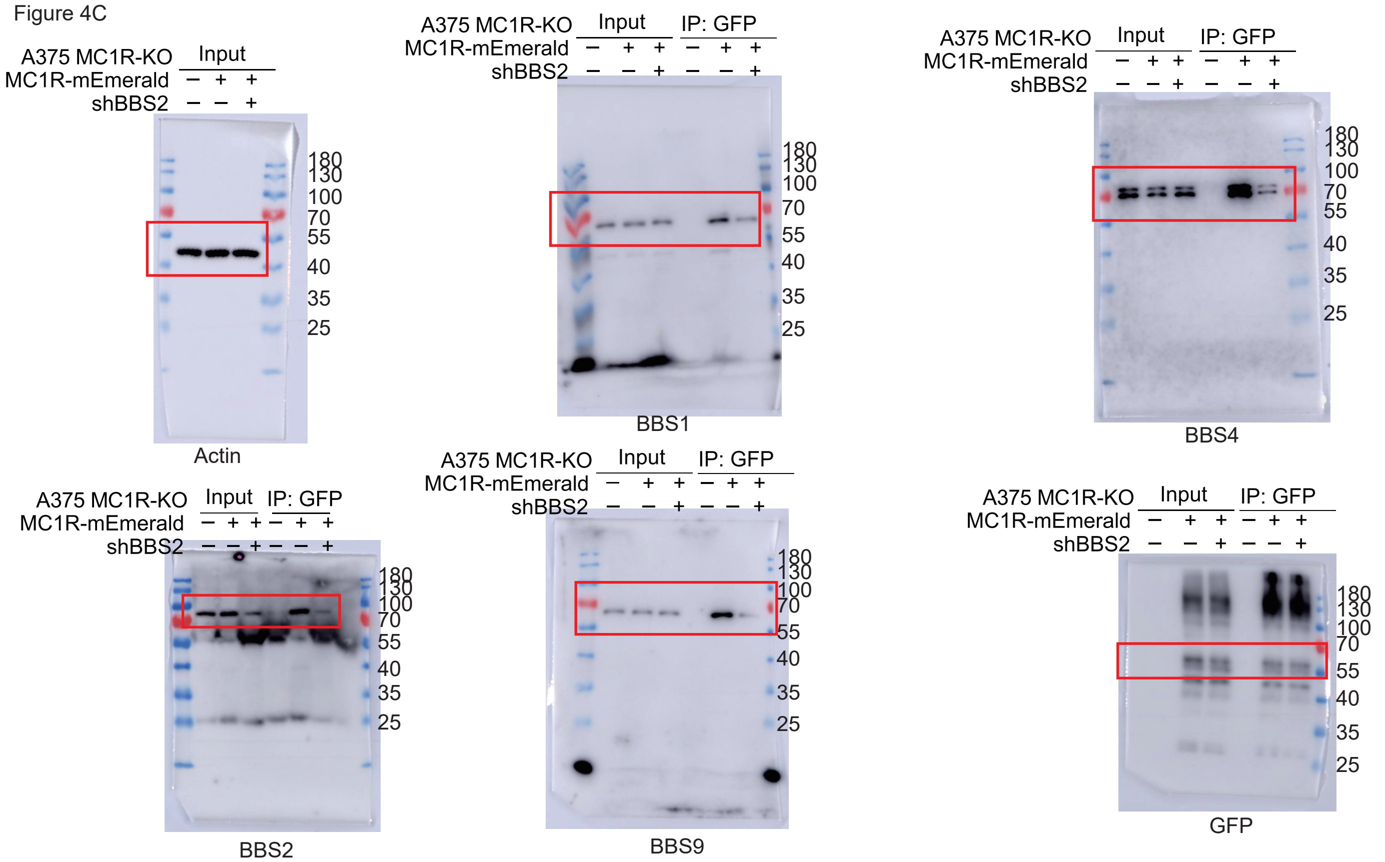

Figure 4D

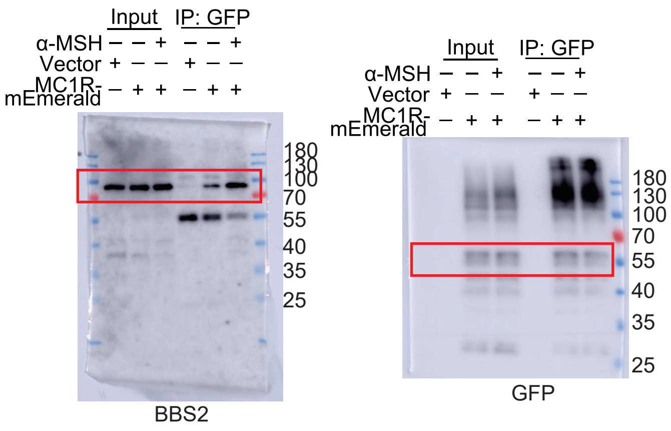

Figure 4E

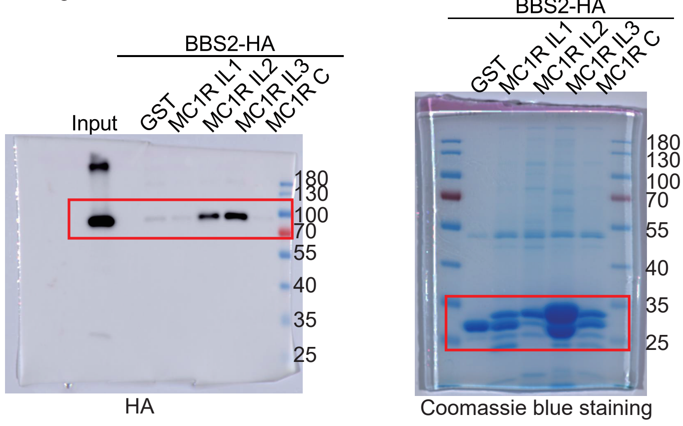

Figure 4F

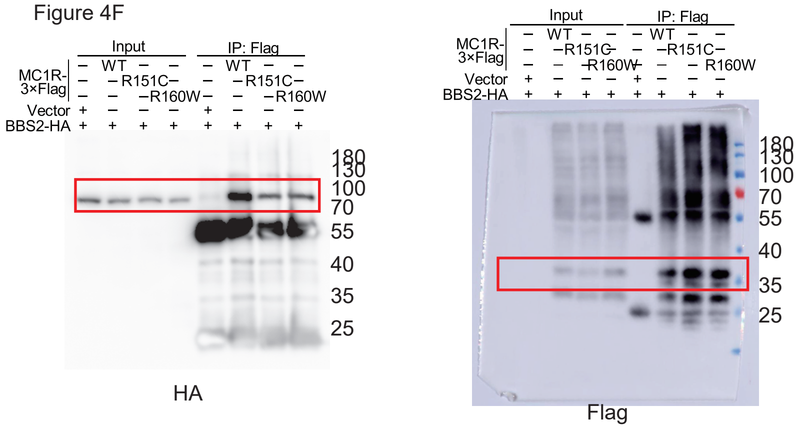

Figure 4K

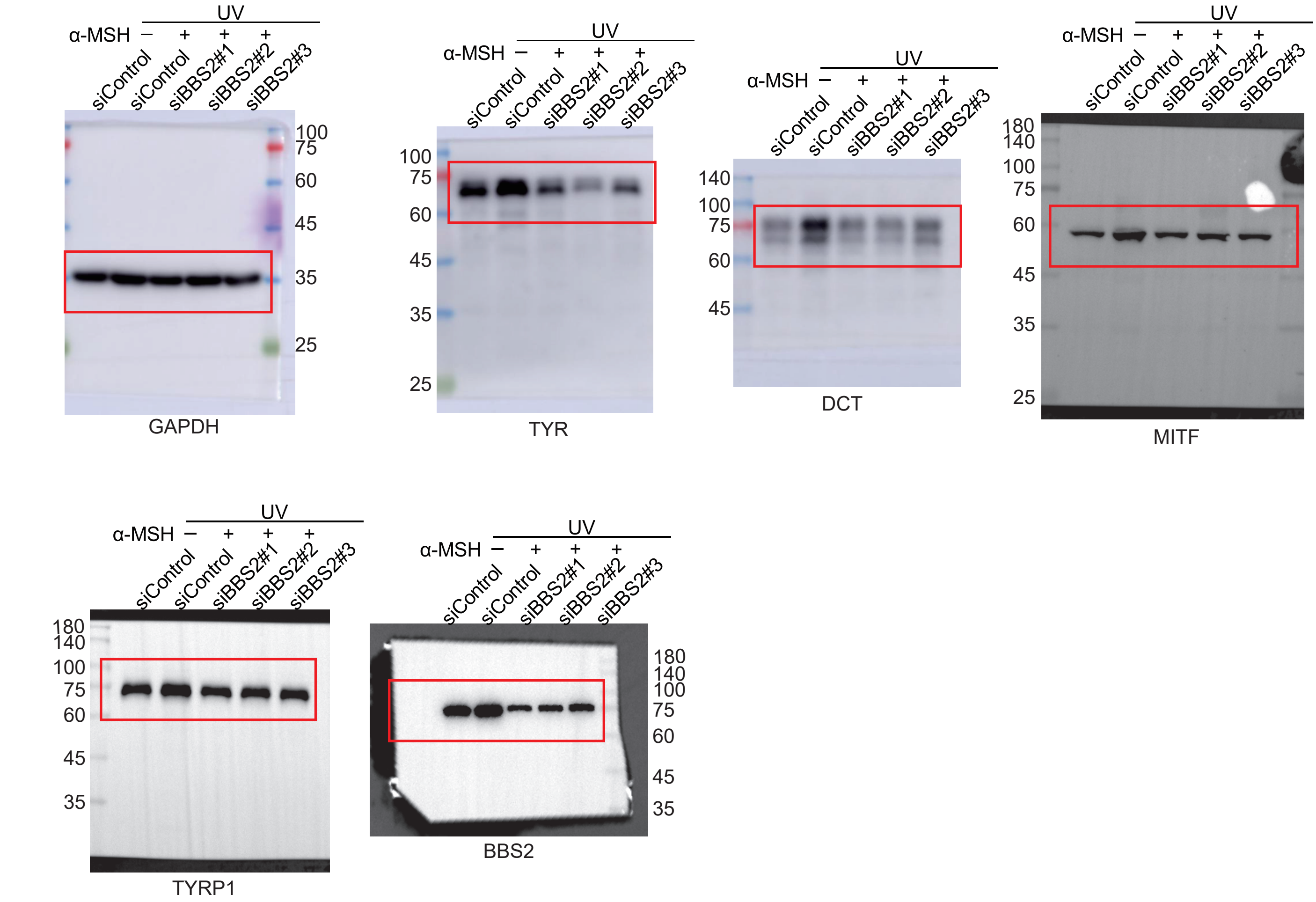

Figure 5E

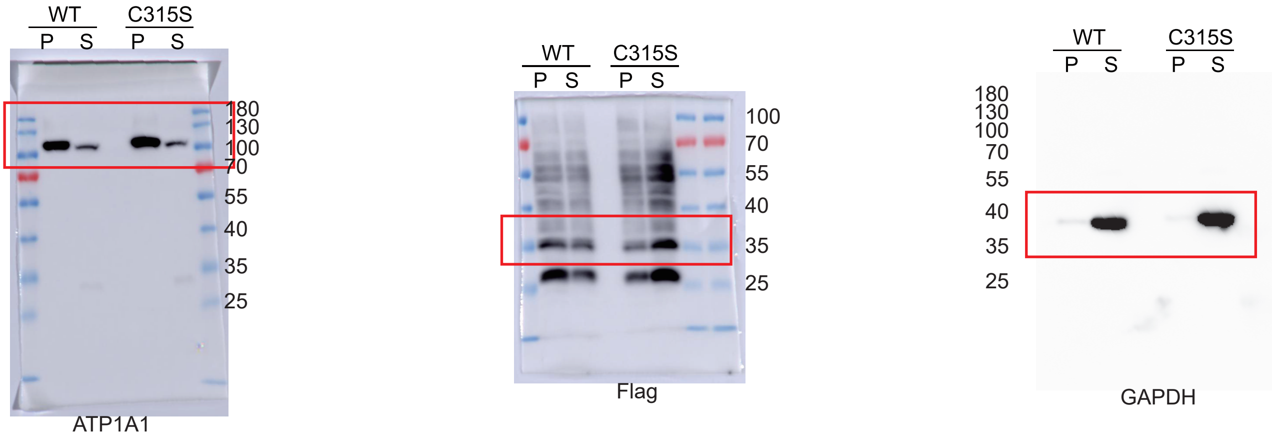

Figure 6F

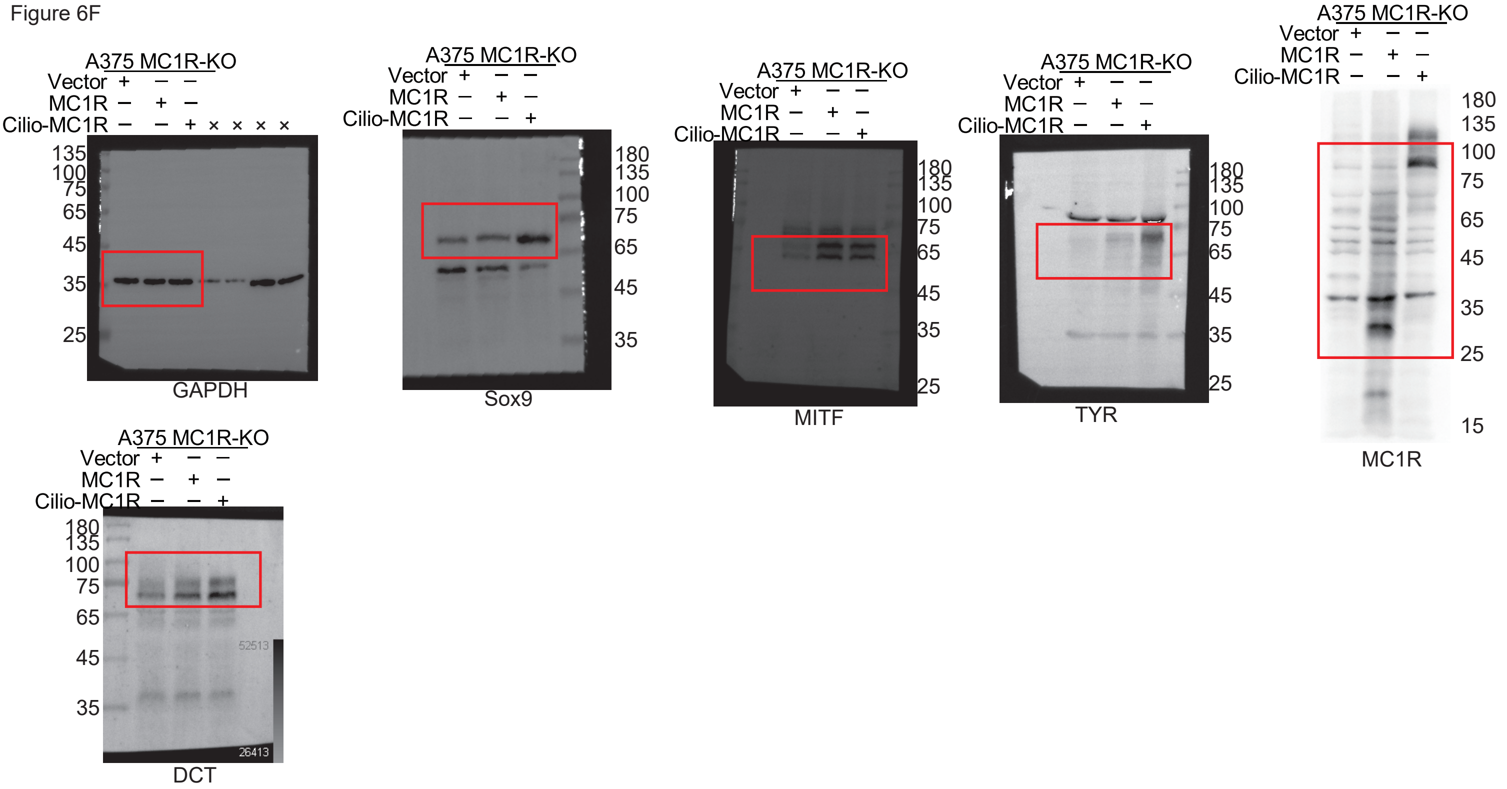

Figure 6G

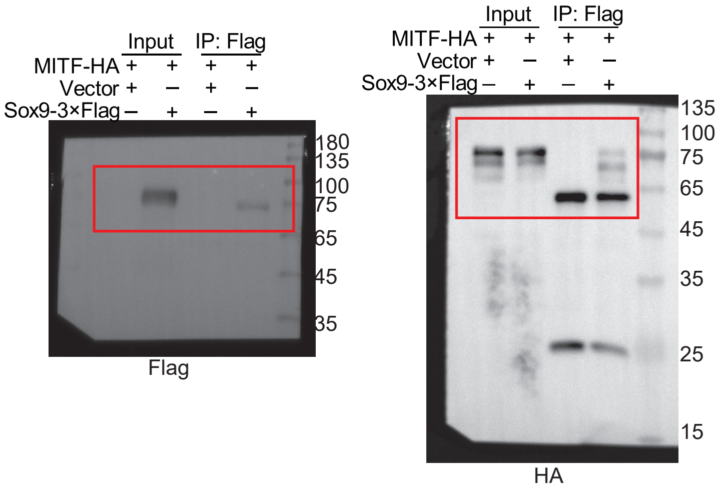

Figure 6H

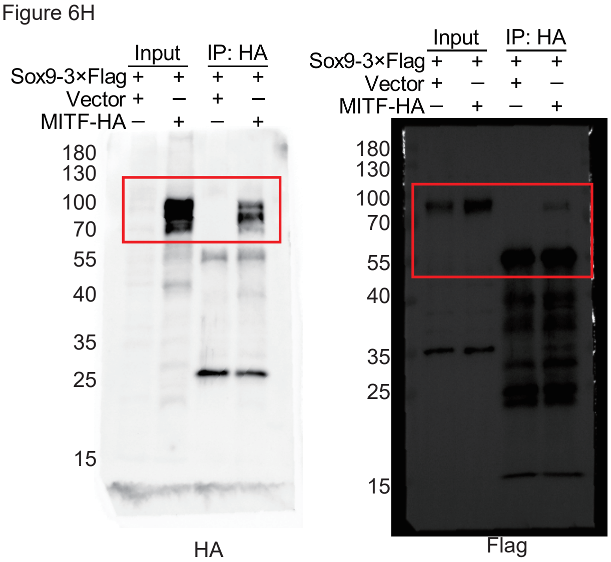

Figure 6K

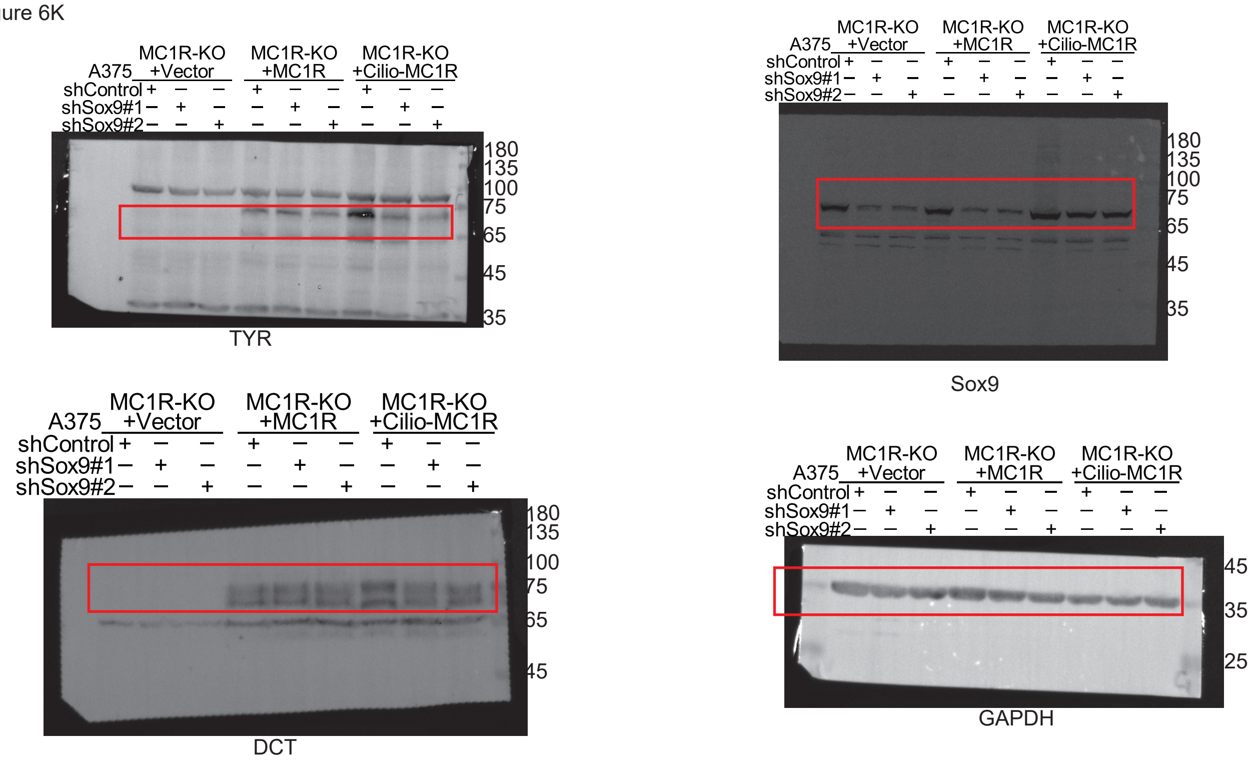

Figure S2A

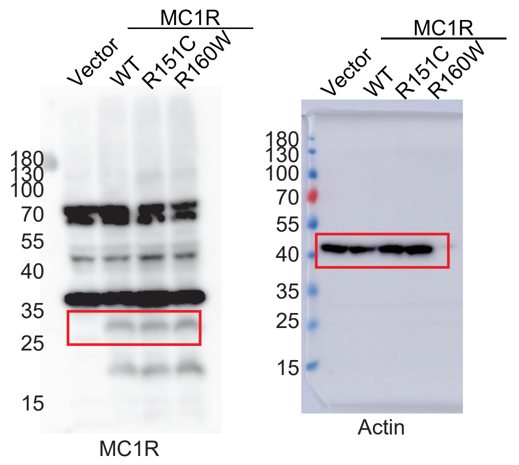

Figure S2D

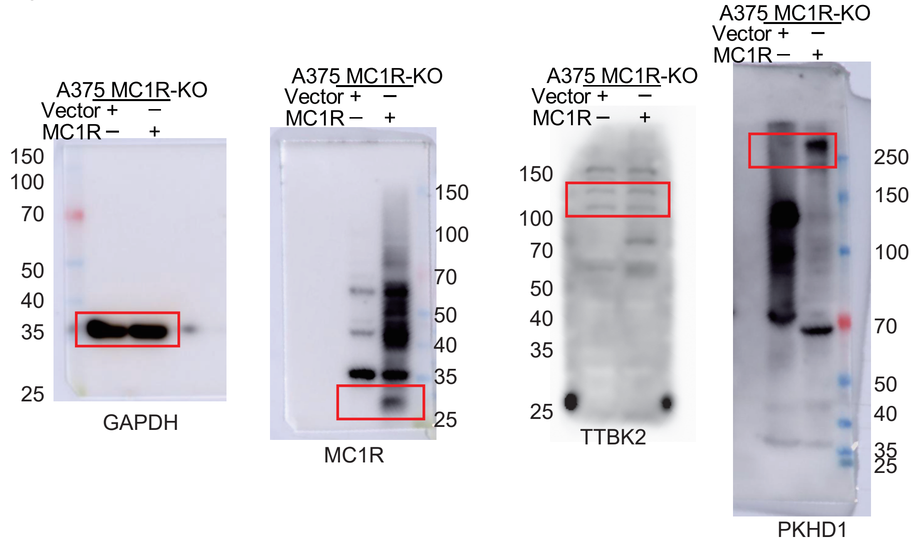

Figure S3G

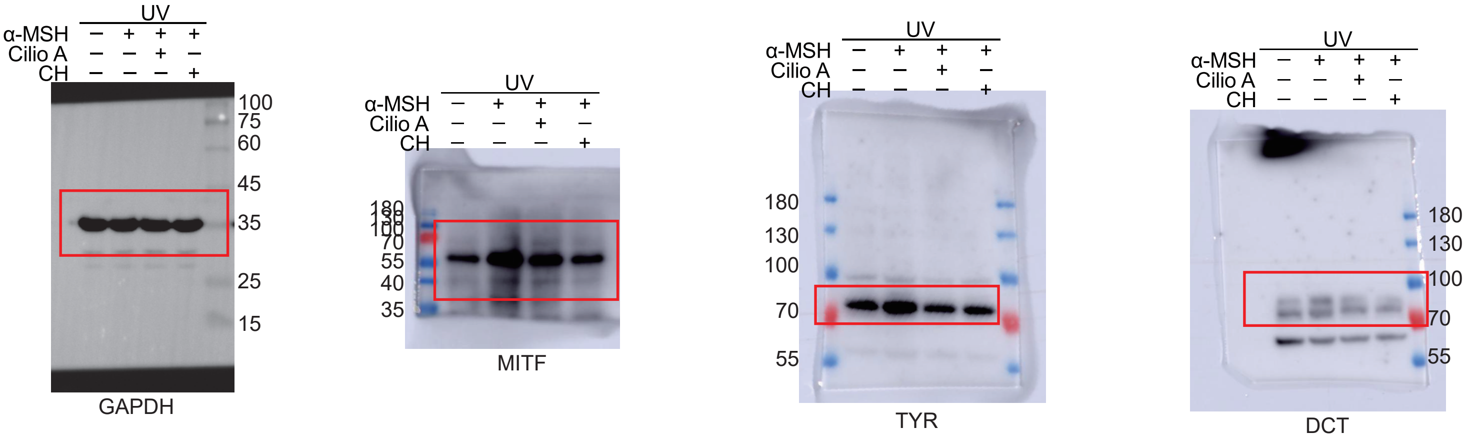

Figure S4G

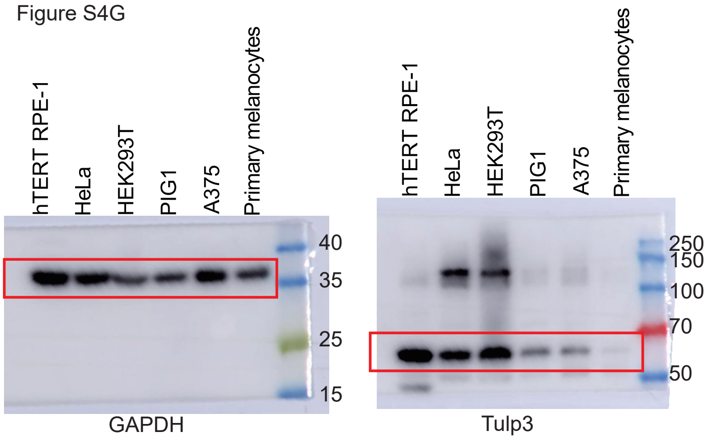

Figure S4H

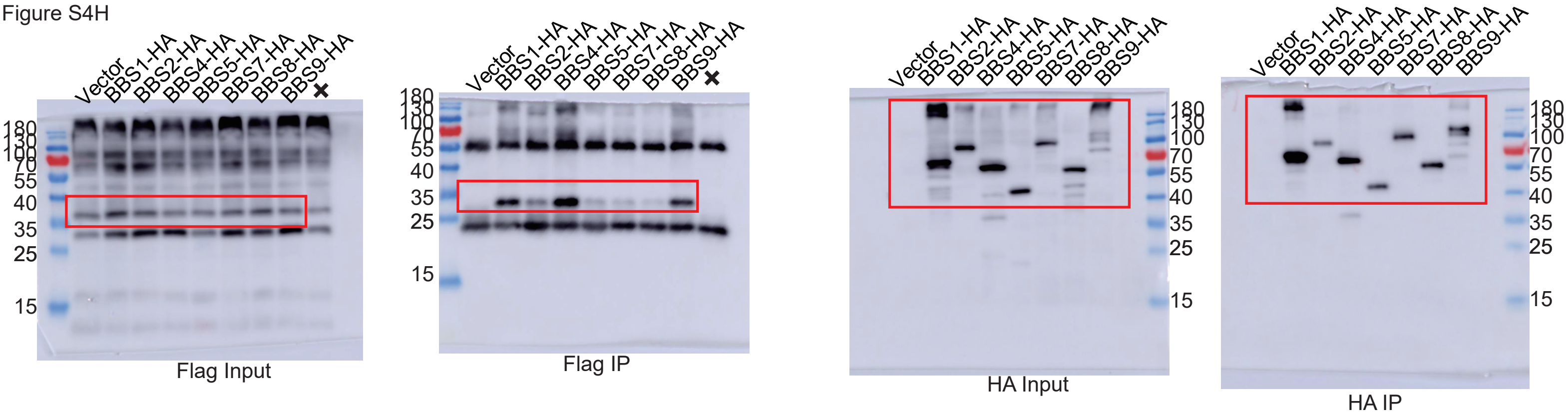

Figure S4J

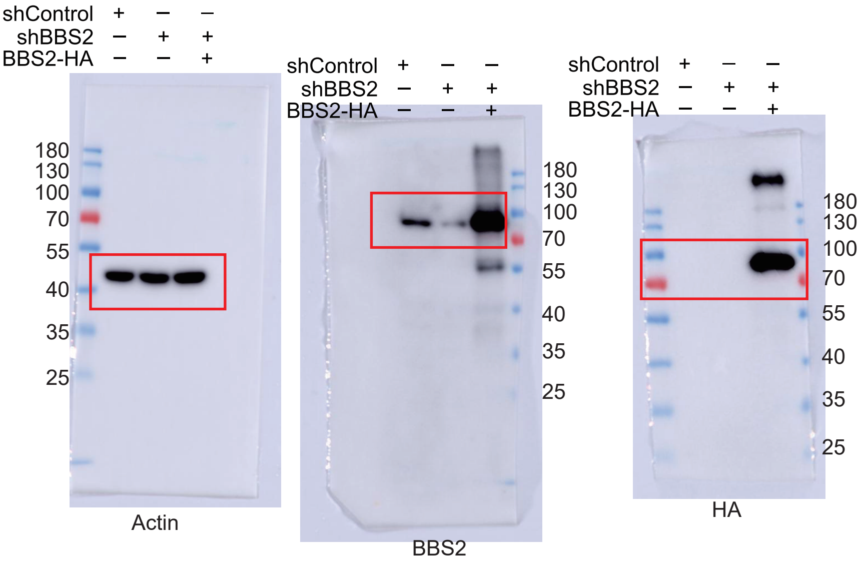

Figure S4M

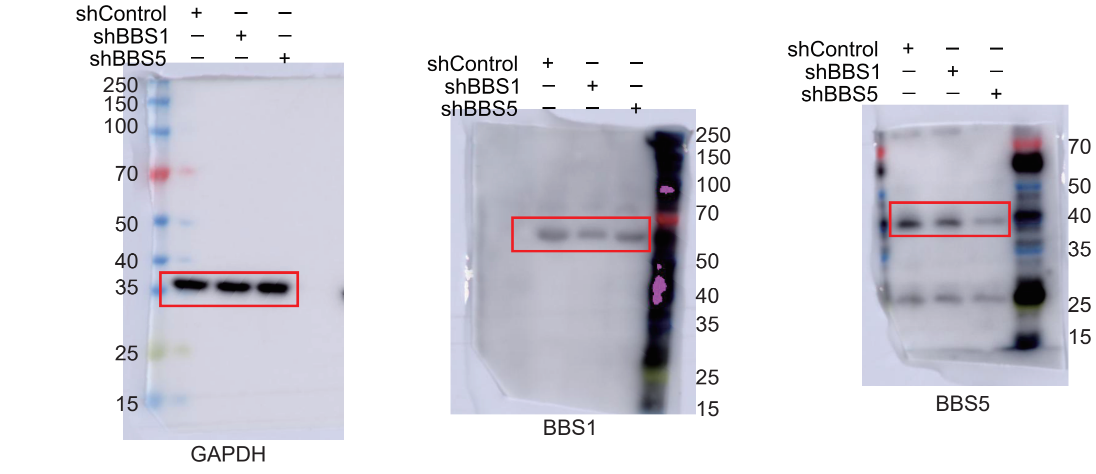

Figure S5A

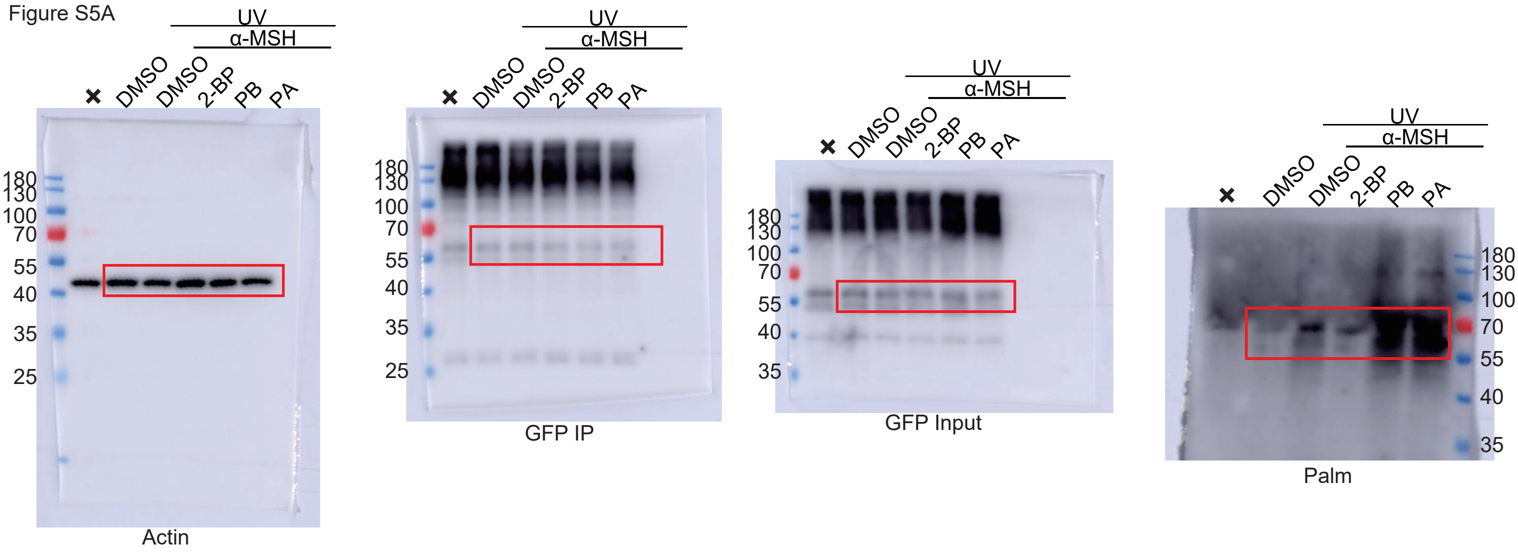

Figure S5B

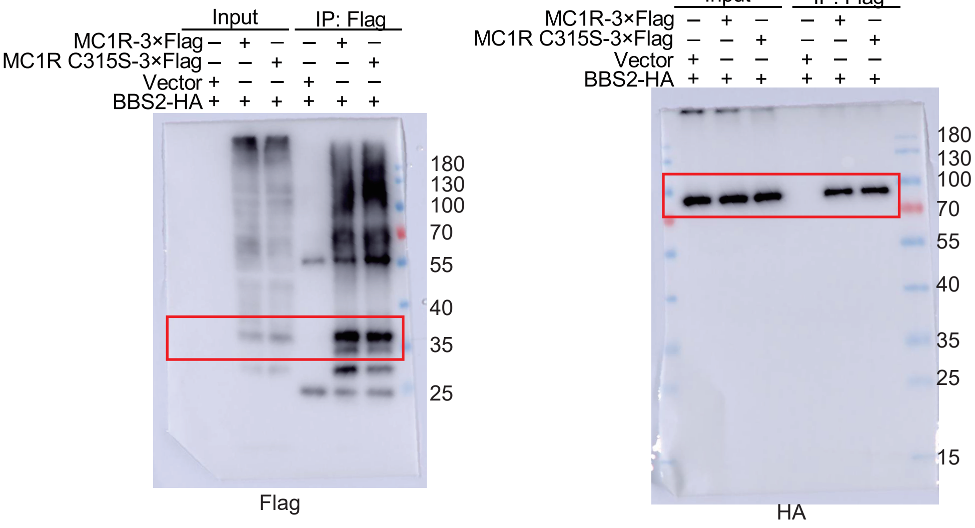

Supplement: S1 Raw Images — (PDF) [file pbio.3002940.s012.pdf]
